# Mechanism of single-stranded DNA annealing by RAD52–RPA complex

**DOI:** 10.1038/s41586-024-07347-7

**Published:** 2024-04-24

**Authors:** Chih-Chao Liang, Luke A. Greenhough, Laura Masino, Sarah Maslen, Ilirjana Bajrami, Marcel Tuppi, Mark Skehel, Ian A. Taylor, Stephen C. West

**Affiliations:** 1 The Francis Crick Institute, London, UK; 2 Present Address: Abcam, Cambridge Biomedical Campus, Cambridge, UK

**Keywords:** Cryoelectron microscopy, DNA, Double-strand DNA breaks

## Abstract

RAD52 is important for the repair of DNA double-stranded breaks^1,2^, mitotic DNA synthesis^3–5^ and alternative telomere length maintenance^6,7^. Central to these functions, RAD52 promotes the annealing of complementary single-stranded DNA (ssDNA)^8,9^ and provides an alternative to BRCA2/RAD51-dependent homologous recombination repair^10^. Inactivation of RAD52 in homologous-recombination-deficient *BRCA1*- or *BRCA2*-defective cells is synthetically lethal^11,12^, and aberrant expression of RAD52 is associated with poor cancer prognosis^13,14^. As a consequence, RAD52 is an attractive therapeutic target against homologous-recombination-deficient breast, ovarian and prostate cancers^15–17^. Here we describe the structure of RAD52 and define the mechanism of annealing. As reported previously^18–20^, RAD52 forms undecameric (11-subunit) ring structures, but these rings do not represent the active form of the enzyme. Instead, cryo-electron microscopy and biochemical analyses revealed that ssDNA annealing is driven by RAD52 open rings in association with replication protein-A (RPA). Atomic models of the RAD52–ssDNA complex show that ssDNA sits in a positively charged channel around the ring. Annealing is driven by the RAD52 N-terminal domains, whereas the C-terminal regions modulate the open-ring conformation and RPA interaction. RPA associates with RAD52 at the site of ring opening with critical interactions occurring between the RPA-interacting domain of RAD52 and the winged helix domain of RPA2. Our studies provide structural snapshots throughout the annealing process and define the molecular mechanism of ssDNA annealing by the RAD52–RPA complex.

## Abstract (web summary)

Single-stranded DNA annealing is driven by RAD52 open rings in association with RPA.

## Main

RAD52 has important roles in two pathways of recombinational repair. The highly conserved N-terminal domain (NTD; amino acids 1–209) binds to ssDNA and promotes ssDNA annealing (SSA)^8,18,19^, while the divergent C-terminal domain interacts with RPA (comprising RPA1, RPA2 and RPA3)^9,21^ and, at least in *Saccharomyces cerevisiae*, promotes the loading of RAD51 recombinase^22–24^. In vertebrates, this latter role is the responsibility of BRCA2^25–27^ and RAD51 paralogue complexes^28,29^, such that the primary role of RAD52 relates to its ability to promote the annealing of complementary ssDNAs. To determine the mechanism of annealing, we used cryo-electron microscopy (cryo-EM) to define the structure of RAD52 and the RAD52–ssDNA complex, and also obtained snapshots of the annealing process through visualization of a RAD52–RPA–ssDNA complex.

## RAD52 open rings promote SSA

**Fig. 1:**
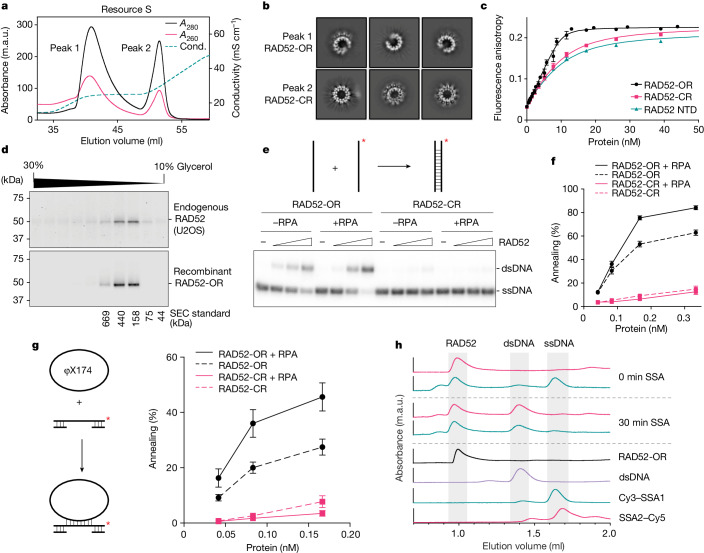
Open rings represent the active form of RAD52. **a**, Resource S cation-exchange chromatography analysis of recombinant human RAD52. Cond., conductivity. **b**, Representative cryo-EM 2D class averages of RAD52 open (RAD52-OR) and closed rings (RAD52-CR). **c**, Single-stranded DNA (40 nucleotides: FAM-SSA4) binding by RAD52-OR, RAD52-CR or RAD52 NTD measured using fluorescence anisotropy. The lines are the best quadratic curve fits. Data are mean + s.e.m. *n* = 6 (RAD52-OR), *n* = 3 (RAD52-CR) and *n* = 3 (RAD52 NTD) independent experiments. **d**, Glycerol gradient sedimentation analysis of a nuclear extract from U2OS cells compared with recombinant RAD52-OR. RAD52 was detected by western blotting. Gel-filtration protein standards are shown. **e**, Representative PAGE assay of SSA by the open or closed rings of RAD52 (0, 0.08, 0.17 and 0.33 nM) using 68-nucleotide-long ssDNA (0.33 nM) with or without RPA (0.33 nM). **f**, Quantification of the SSA assays from **e**. Data are mean + s.e.m. *n* = 22 (RAD52-OR and RAD52-OR + RPA) and *n* = 7 (RAD52-CR and RAD52-CR + RPA) independent experiments. **g**, SSA using φX174 circular ssDNA and a gapped duplex by RAD52 (OR or CR) in the presence or absence of RPA. Data are mean + s.e.m. *n* = 4 independent experiments. **h**, SEC analysis of RAD52-mediated SSA between Cy3–SSA1 (dark cyan, recorded at 647 nm) and SSA2–Cy5 (pink, 555 nm) labelled ssDNAs. RAD52 was preloaded on SSA2–Cy5 before addition of Cy3–SSA1. RAD52-OR (black) was recorded at 280 nm. In **e**,**g**, ^32^P labels are indicated with asterisks.

**Extended Data Fig. 1:**
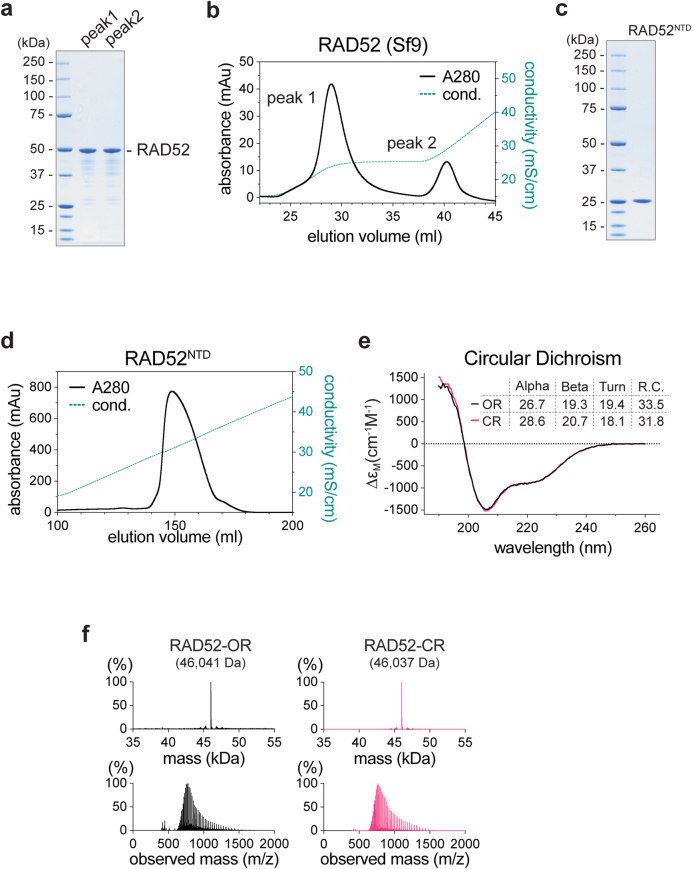
Characterization of human RAD52. **a**, Coomassie blue stained SDS-PAGE of the two RAD52 peaks from Resource S cation exchange chromatography (as Fig. 1a). **b**, Resource S chromatography of human _FLAG_RAD52 expressed in Sf9 insect cells. **c**, SDS-PAGE gel showing RAD52^NTD^ (1-209aa). **d**, Resource S chromatography of RAD52^NTD^. **e**, Far-UV circular dichroism (CD) spectra of RAD52 open (OR) and closed rings (CR). Secondary structure content estimates obtained by spectral deconvolution are indicated. **f**, Intact protein mass spectrometry of RAD52-OR and RAD52-CR. Upper panels: deconvoluted spectra; lower panels: raw spectra. The measured mass of each protein is shown.

Human RAD52 was expressed in *Escherichia coli* and purified to homogeneity. During cation-exchange chromatography, the protein separated into two distinct species that appeared identical when analysed by SDS–PAGE (Fig. 1a and Extended Data Fig. 1a). Similar results were obtained with protein expressed in baculovirus-infected Sf9 insect cells (Extended Data Fig. 1b). By contrast, the N-terminal domain (NTD) of RAD52^18,19^ eluted as a single species from this column (Extended Data Fig. 1c,d). When visualized using cryo-EM, we found that the two species of full-length RAD52 represent open (RAD52-OR, peak 1) and closed (RAD52-CR, peak 2) ring forms (Fig. 1b). The 11-subunit closed rings exhibit features similar to the RAD52 NTD, whereas the RAD52 open rings have one or more subunits missing. Circular dichroism confirmed that both forms adopted the same secondary structure (Extended Data Fig. 1e), and intact protein mass spectrometry (MS) analysis showed that their mass approximated the calculated mass of RAD52 less the N-terminal methionine residue (Extended Data Fig. 1f).

**Extended Data Fig. 2:**
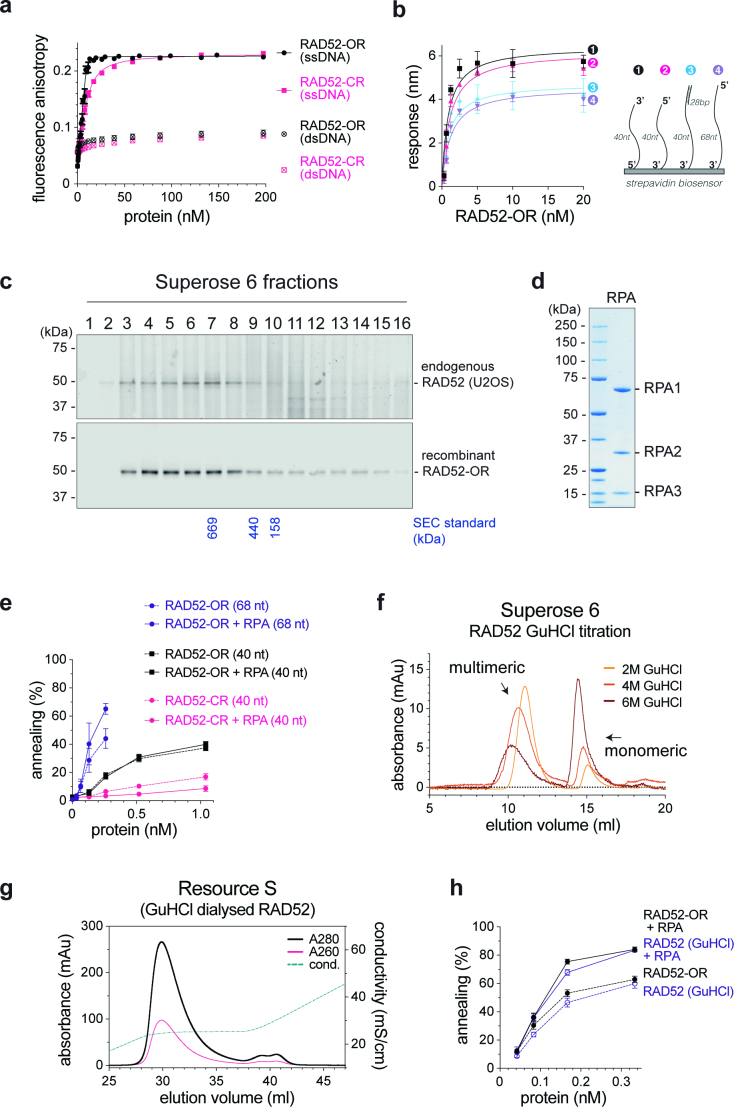
Single strand DNA annealing by RAD52. **a**, RAD52-OR and RAD52-CR binding to 40 nt long ssDNA (10 nM, FAM-SSA4) or dsDNA (10 nM, FAM-SSA4/SSA5) measured by fluorescence anisotropy. Lines denote best quadratic curve fits. Each point and error bar denotes mean + s.e.m. (RAD52-OR-ssDNA, n = 6; RAD52-CR-ssDNA, n = 3; RAD52-OR-dsDNA, n = 3; RAD52-CR-dsDNA, n = 3). n values are independent experiments. **b**, RAD52-OR binding to the indicated biotinylated DNAs, as measured by biolayer interferometry. Lines denote 1:1 binding curve fits. Each point and error bar denotes mean + s.e.m. (n = 3). Equilibrium dissociation constants (*K*_D_) were: (1) 0.89 ± 0.35 nM, (2) 1.11 ± 0.4 nM, (3) 1.0 ± 0.55 nM and (4) 1.23 ± 0.41 nM. **c**, Size exclusion chromatography of a nuclear extract from U2OS cells compared with recombinant RAD52-OR. RAD52 was detected by Western blotting. **d**, SDS-PAGE of RPA. **e**, SSA using RAD52 (OR or CR) in the presence or absence of RPA with 68 or 40 nt ssDNA. Each point and error bar denotes mean + s.e.m. (RAD52-OR [68 nt] and RAD52-OR + RPA [68 nt], n = 3; RAD52-OR [40 nt] and RAD52-OR + RPA [40 nt], n = 9; RAD52-OR [40 nt] and RAD52-OR + RPA [40 nt], n = 3). n values are independent experiments. **f**, Superose 6 filtration of RAD52 following dialysis in 2, 4 and 6 M GuHCl. **g**, Resource S chromatography of RAD52 following GuHCl denaturation and refolding. **h**, SSA using renatured RAD52, as in Fig. 1f. Each point and error bar denotes mean + s.e.m. (RAD52-OR and RAD52-OR + RPA, n = 22; RAD52-OR [GuHCl] and RAD52-OR [GuHCl] + RPA, n = 3). n values are independent experiments.

The RAD52 open rings exhibited a high affinity (*K*_D_ = 0.3 ± 0.1 nM) for 40-nucleotide-long ssDNA but not double-stranded DNA (dsDNA), as measured by fluorescence anisotropy (Fig. 1c and Extended Data Fig. 2a). Binding was observed with linear ssDNA, ssDNAs in which the 5′ or 3′ ends were blocked by biotin–streptavidin, and a ssDNA/dsDNA substrate, as measured using biolayer interferometry (Extended Data Fig. 2b). By contrast, the RAD52 closed rings bound to ssDNA with reduced affinity (3.3 ± 0.5 nM) comparable to that of the RAD52 NTD (3.2 ± 0.6 nM) (Fig. 1c).

To determine whether the RAD52 oligomers, purified after overexpression in *E. coli* or insect cells, are representative of RAD52 within human cells, the recombinant RAD52 open rings were compared with endogenous RAD52 contained within a nuclear extract from U2O2 cells. Endogenous RAD52 exhibited a similar oligomeric state to the recombinant RAD52-ORs, as determined using glycerol gradient sedimentation (Fig. 1d) and size-exclusion chromatography (Extended Data Fig. 2c).

We next analysed single-strand annealing by RAD52 using in vitro assays in which ^32^P-labelled ssDNA (68 nucleotides) was incubated with its complementary strand in the presence or absence of RPA (Fig. 1e and Extended Data Fig. 2d). The RAD52-ORs annealed ssDNA in reactions stimulated by RPA, whereas the RAD52-CRs exhibited a reduced ability to promote annealing that was unaffected by the presence or absence of RPA (Fig. 1e,f). The stimulatory effect of RPA on RAD52-OR-mediated annealing was not observed with shorter ssDNAs (40 nucleotides) (Extended Data Fig. 2e). As a RAD52 ring can bind to approximately 40 nucleotides of ssDNA^18,30^, these results show that efficient annealing requires the stable association of both RAD52 and RPA on ssDNA. Consistent with the DNA-binding experiments, RAD52-OR-mediated SSA did not require free ssDNA ends (Fig. 1g). Size-exclusion chromatography (SEC) revealed that dsDNA dissociated from RAD52 after completion of annealing (Fig. 1h), consistent with the low affinity of RAD52 for dsDNA (Extended Data Fig. 2a).

Denaturation of a mixed population of open and closed rings of RAD52 using guanidinium hydrochloride, followed by renaturation, resulted in the majority of the protein adopting an open-ring conformation (Extended Data Fig. 2f,g). The refolded RAD52-ORs were as active as purified RAD52 open rings (Extended Data Fig. 2h), consistent with the observation that open rings represent the active form of RAD52.

## RAD52-CR and RAD52-OR structures

**Fig. 2:**
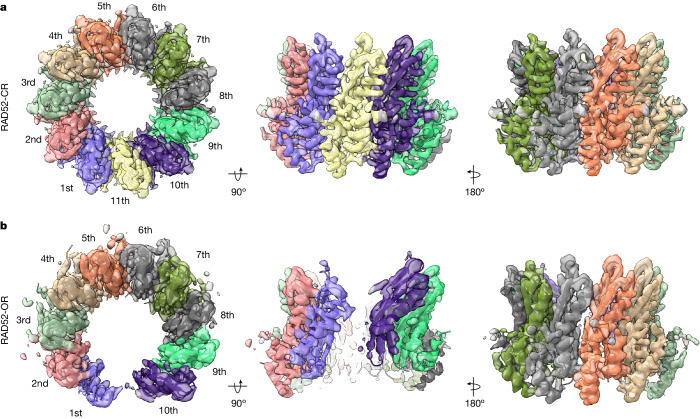
Cryo-EM structures of RAD52 closed and open rings. **a**, Top and side views of the RAD52-CR cryo-EM map (2.9 Å) and atomic model. **b**, Top and side views of the RAD52-OR cryo-EM map (3.2 Å) and atomic model. RAD52 subunits are numbered successively.

**Extended Data Fig. 3:**
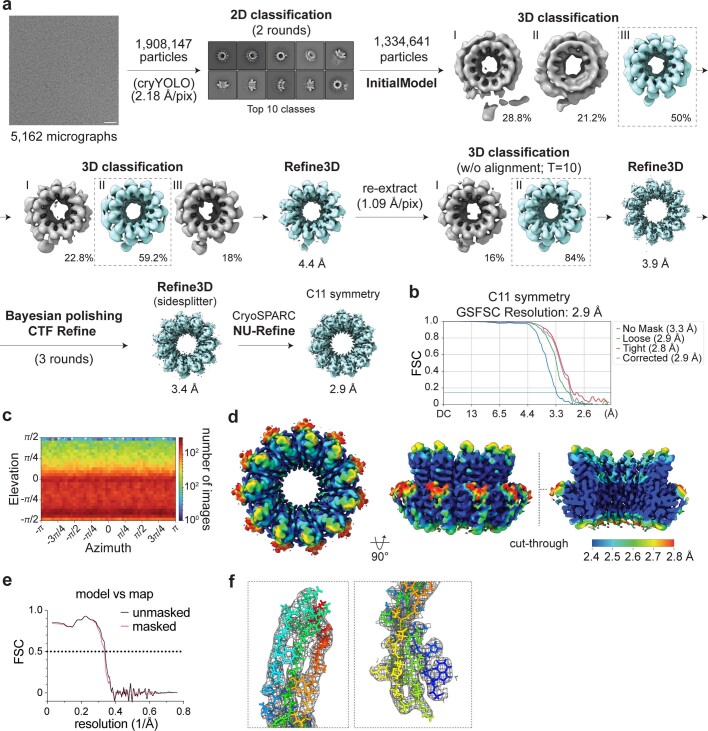
Cryo-EM data processing of RAD52-CR. **a**, Schematic showing the classification and refinement steps to determine the cryo-EM structure of RAD52-CR. The software used during each processing step is indicated if outside of RELION. Scale bar for representative micrograph = 50 nm. **b**, Fourier Shell Correlation (FSC) plot for C11 symmetry refinement. **c**, Angular distribution plot for C11 symmetry refinement. **d**, Top, side and cut-through views of RAD52-CR cryo-EM density (C11 symmetry) coloured by local resolution estimated by CryoSPARC. **e**, Model to map correlation graph. **f**, Zoom view of RAD52-CR cryo-EM map and atomic model focusing on 83-133aa of the 5^th^ RAD52 subunit.

**Extended Data Fig. 4:**
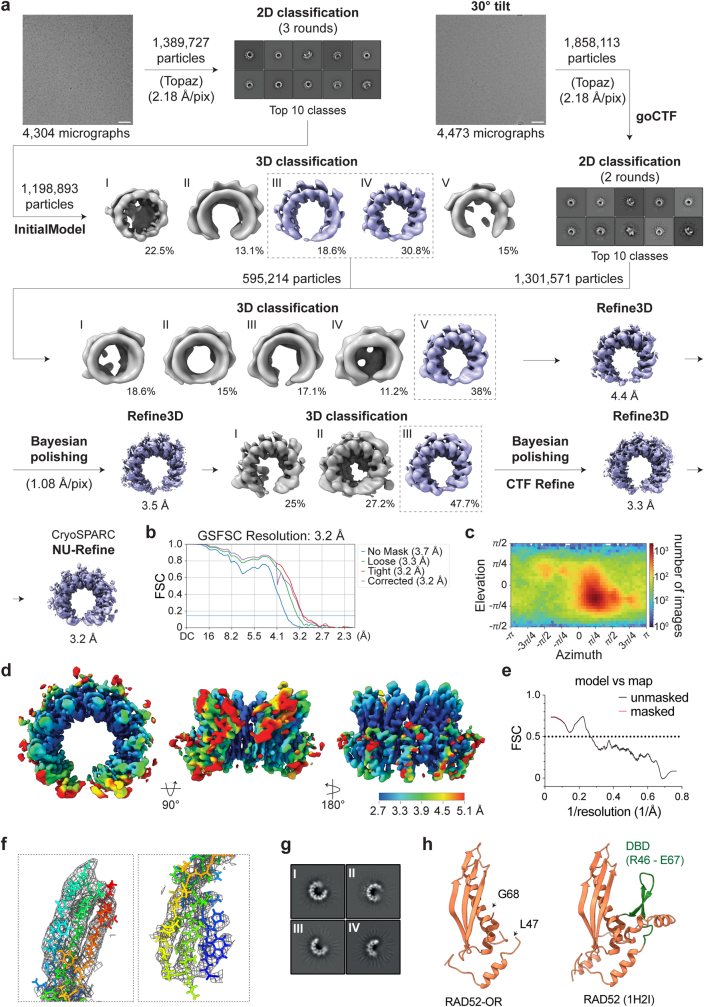
Cryo-EM data processing of RAD52-OR. **a**, As Extended Data Fig. 3, except with the RAD52-OR. **b**, Fourier Shell Correlation (FSC) plot. **c**, Angular distribution plot. **d**, Top, front and back views of RAD52-OR cryo-EM density coloured by local resolution estimated by CryoSPARC. **e**, Model to map correlation graph. **f**, Zoom view of RAD52-OR cryo-EM map and atomic model focusing on 83-133aa of the 5^th^ RAD52 subunit. **g**, Representative cryo-EM EM 2D averages of RAD52 open ring with (I) 9, (II) 7 – 8, (III) 6 and (IV) 5 subunits. **h**, Comparison of a RAD52 protomer from 1H2I crystal structure with the open ring cryo-EM structure.

The structures of RAD52-CR (2.9 Å) and RAD52-OR (3.2 Å) were determined using cryo-EM (Fig. 2, Extended Data Figs. 3a–f and 4a–f and Extended Data Table 1). The closed ring comprised 11 subunits as observed previously^18,19,31^. The RAD52-OR structures contained a maximum of ten subunits, although open rings with fewer subunits were also observed (Extended Data Fig. 4a,g). The structure of each subunit in the open ring was similar to that of the closed ring. As expected, the cryo-EM density around the site of ring opening was not as well resolved as other parts of the structure (Extended Data Fig. 4d).

## Interaction between RAD52 and ssDNA

**Fig. 3:**
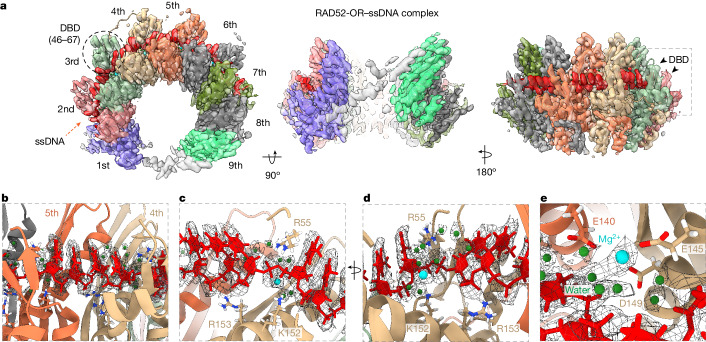
Cryo-EM structure of the RAD52–ssDNA complex. **a**, Top and side views of the RAD52–ssDNA complex cryo-EM map (2.3 Å) and atomic model. ssDNA is coloured red. The DBD is indicated on the third RAD52 subunit. **b**, Magnified view showing four ssDNA nucleotides (red) adopting the length of B-form DNA between two RAD52 monomers. **c**,**d**, Magnified view of ssDNA binding by Arg55, Lys152 and Arg153. **e**, Magnified view showing Mg^2+^ (cyan) and water molecules (green) coordinated by Glu140 (from a neighbouring RAD52 subunit), Glu145 and Asp149. The cryo-EM densities of ssDNA, Mg^2+^ and water molecules are presented as a mesh.

**Extended Data Fig. 5:**
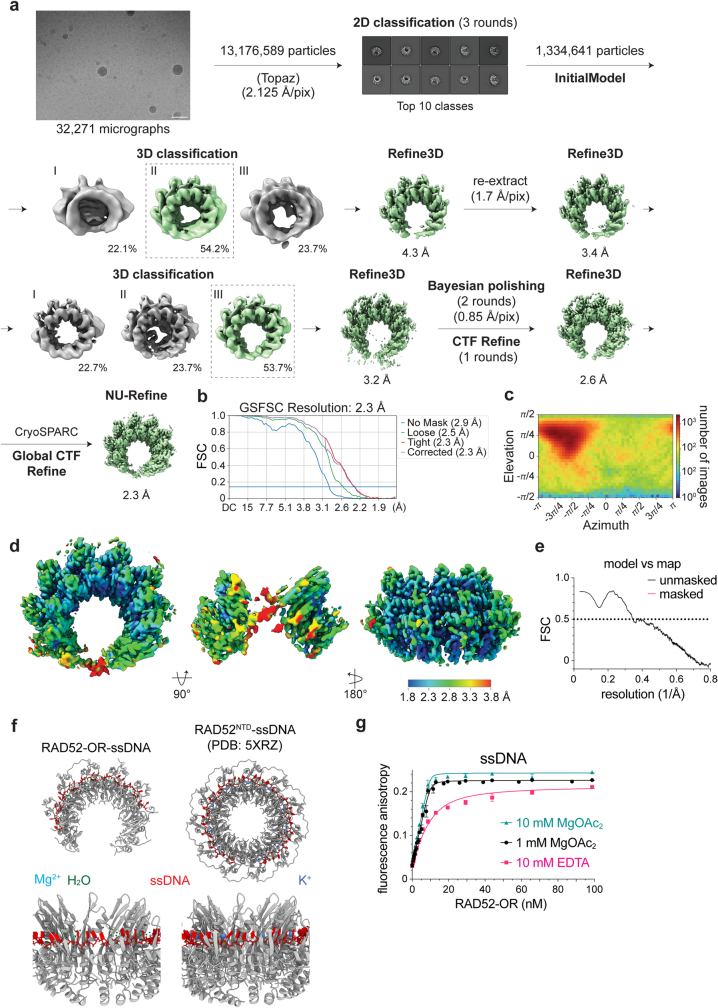
Cryo-EM data processing of RAD52-ssDNA complex. **a**, As Extended Data Fig. 3, except for the RAD52-OR-ssDNA structure. **b**, Fourier Shell Correlation (FSC) plot. **c**, Angular distribution plot. **d**, Top, front and back views of the RAD52-OR-ssDNA cryo-EM density coloured by local resolution estimated by CryoSPARC. **e**, Model to map correlation graph. **f**, Comparison between RAD52-OR-ssDNA and RAD52^NTD-K102A/K133A^-ssDNA (PDB: 5XRZ) structures. **g**, Fluorescence anisotropy of RAD52-OR binding to ssDNA (FAM-SSA4; 40 nt) at different Mg^2+^ concentrations. Lines are best quadratic curve fits. Each point and error bar denotes mean + s.e.m. (n = 3). n values are independent experiments.

In contrast to the X-ray crystal structure of the RAD52 NTD (Protein Data Bank (PDB): 1H2I)^18,19^, we could not visualize the ssDNA-binding domain (DBD, amino acids 46–67) in the cryo-EM model of full-length RAD52 (Extended Data Fig. 4h). One possibility is that this domain is flexible before interaction with ssDNA, enabling rapid and tight association with ssDNA (Fig. 1c). To determine the validity of this hypothesis, the structure of the RAD52-OR bound to ssDNA (2.3 Å) was solved (Fig. 3a, Extended Data Fig. 5a–e and Extended Data Table 1). An analysis of the RAD52–ssDNA complex revealed that the DBD was stabilized as ssDNA bound into the positively charged groove on the outside of the RAD52 ring. A comparison with the crystal structure of the RAD52-NTD(K102A/K133A)–ssDNA complex (PDB: 5XRZ) revealed a very similar organization of the ssDNA (Extended Data Fig. 5f).

In the RAD52-OR–ssDNA complex, the ssDNA could be visualized from the second to the seventh RAD52 subunit (Fig. 3a). There were four ssDNA nucleotides per RAD52 subunit (Fig. 3b), consistent with the observed four-nucleotide pattern of hypersensitivity to hydroxyl radicals^30^. Topological analysis of the structure revealed that the bound ssDNA was stretched non-uniformly, such that each quartet stack adopted the length of B-form duplex DNA. Arg153 coordinated the phosphate backbone of the two central nucleotides (Fig. 3c), whereas Arg55 and Lys152 coordinated the phosphate backbone between two quartet stacks (Fig. 3d). The Mg^2+^, which is required for RAD52–ssDNA interactions (Extended Data Fig. 5g), and the surrounding water molecules were coordinated by Glu145, Asp149 and Glu140 residues from the neighbouring subunit (Fig. 3e).

## Oligomeric state of human RAD52

**Fig. 4:**
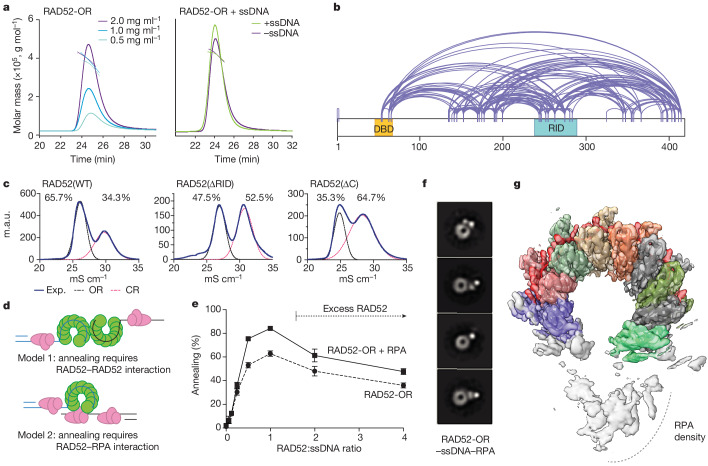
Mechanisms of annealing by RAD52. **a**, SEC coupled to multi-angle laser light scattering (SEC–MALLS) analysis measuring the molecular mass of RAD52-OR at different protein concentrations and in the presence or absence of ssDNA. The solid lines are the chromatograms from the output of the differential refractometer and the scatter points are the weight-averaged molar masses determined at 1 s intervals throughout elution of the chromatographic peaks. **b**, XL-MS analysis of RAD52-ORs. **c**, Resource S cation chromatography of WT RAD52, RAD52(∆RID) and RAD52(∆C). Experimental ultraviolet 280 nm (UV_280_) absorbance is depicted as a solid blue line. Deconvoluted peaks are indicated (black lines, open ring; pink lines, closed ring). **d**, Schematic of the two possible mechanisms for single-stranded annealing: (1) SSA by interactions between two complementary RAD52-bound ssDNAs; and (2) SSA involving interactions between the RAD52–ssDNA complex and complementary-strand ssDNA bound by RPA. **e**, The effect of RAD52 concentration on ssDNA (0.33 nM; 68 nucleotides) annealing in the presence and absence of RPA (0.33 nM). Data are mean + s.e.m. *n* = 5 (RAD52-OR and RAD52-OR + RPA) independent experiments. **f**, Representative negative-stain EM 2D averages of reconstituted RAD52-OR–ssDNA–RPA complex (ssDNA: 68 nucleotides). **g**, Top view of the RAD52-OR–ssDNA–RPA complex cryo-EM map (3 Å) with the RAD52–ssDNA (68 nucleotides) atomic model.

Previously, undecameric and heptameric RAD52 closed rings were observed^18,19,32^, indicating a degree of structural flexibility by which subunit–subunit interactions can accommodate changes to the overall size of the protein. Consistent with this, in addition to the ten-subunit open ring, our analysis of two-dimensional (2D) averages and three-dimensional (3D) classes indicated the presence of RAD52-OR structures with fewer subunits (Extended Data Fig. 4a,g). To determine whether the oligomeric state was influenced by protein concentration, size-exclusion chromatography coupled with multi-angle laser light scattering (SEC-MALLS) was used to determine the molecular mass of RAD52-OR at a variety of concentrations and the in presence or absence of ssDNA. We found that the oligomeric state was dependent on protein concentration but was unaffected by ssDNA binding (Fig. 4a).

## Interactions modulate ring dynamics

**Extended Data Fig. 6:**
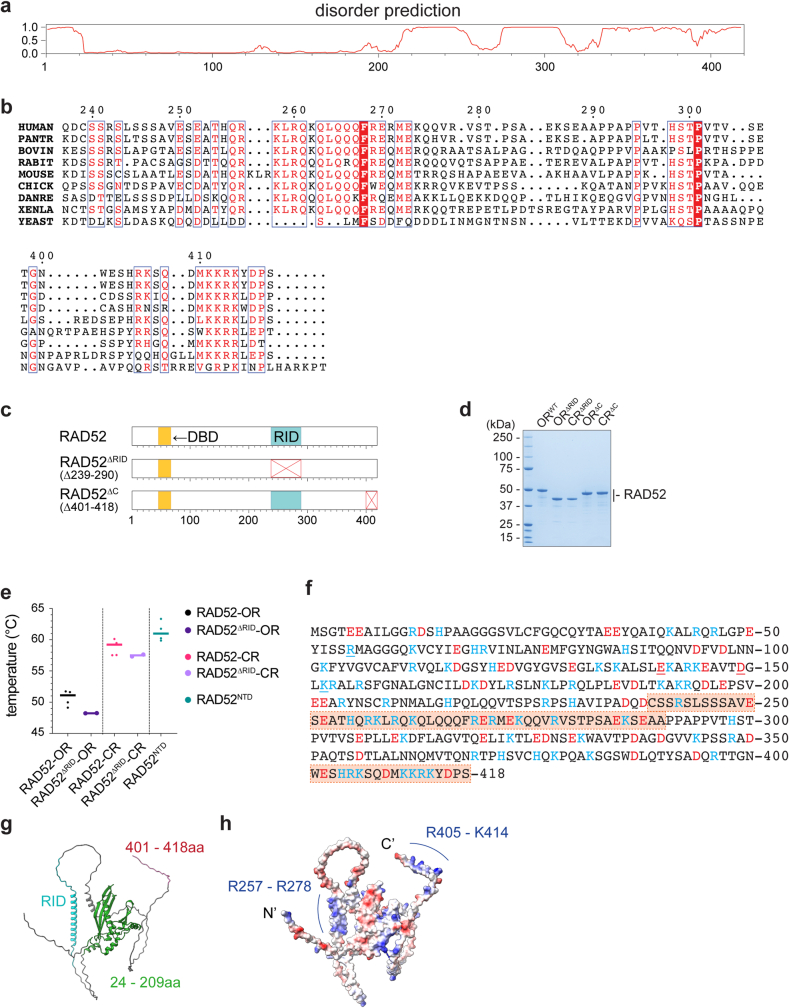
Analysis of RAD52 mutants. **a**, Protein disorder prediction of human RAD52. **b**, Clustal Omega sequence alignment reveals conservation of 239-290 and 401-418aa in vertebrates. **c**, Domain architecture of RAD52, and the RAD52^∆RID^ and RAD52^∆C^ deletion mutants. **d**, SDS-PAGE of RAD52^WT^-OR, RAD52^∆RID^-OR, RAD52^∆RID^-CR, RAD52^∆C^-OR and RAD52^∆C^-CR, stained with Coomassie blue. **e**, Thermal melting analysis of the indicated proteins. Each point and error bar denotes mean + s.d. RAD52^WT^-OR (50.7 ± 1.1 °C; n = 5), RAD52^∆RID^-OR (48.2 ± 0.01 °C; n = 2), RAD52^WT^-CR (58.8 ± 1.2 °C; n = 5), RAD52^∆RID^-CR (57.5 ± 0.2 °C; n = 2) and RAD52^NTD^ (61.3 ± 1.6 °C; n = 4). n values are independent experiments. **f**, Human RAD52 amino acid sequence coloured by electrostatic potential (blue, positively charged; red, negatively charged). Boxed and shaded sequence highlights the RAD52 RID (239-290aa) and the extreme C-terminus (401-418aa). **g**, AlphaFold2 prediction of RAD52 structure. The N-terminal domain (24-209aa, green), RID (239-290aa, cyan) and extreme C-terminus (401-418aa, red) are indicated. **h**, AlphaFold2 predicted RAD52 structure shown in surface view coloured with electrostatic potential.

The C-terminal region of RAD52 (amino acids 210–418) is predicted to be structurally disordered (Extended Data Fig. 6a). However, it is an important part of RAD52 as it interacts with RPA^21,33^ and is required for nuclear localization^34,35^. As this region was not resolved in the cryo-EM structures, we used cross-linking MS (XL-MS) to detect dynamic interactions^36^ between the N- and C-terminal domains of RAD52 (Fig. 4b). The N-terminal domain interacted with three hotspots in the C terminus of RAD52: one mapped to the RPA-interacting domain (RID), one is located around amino acid 35, and the other was located at the extreme C terminus.

**Extended Data Fig. 7:**
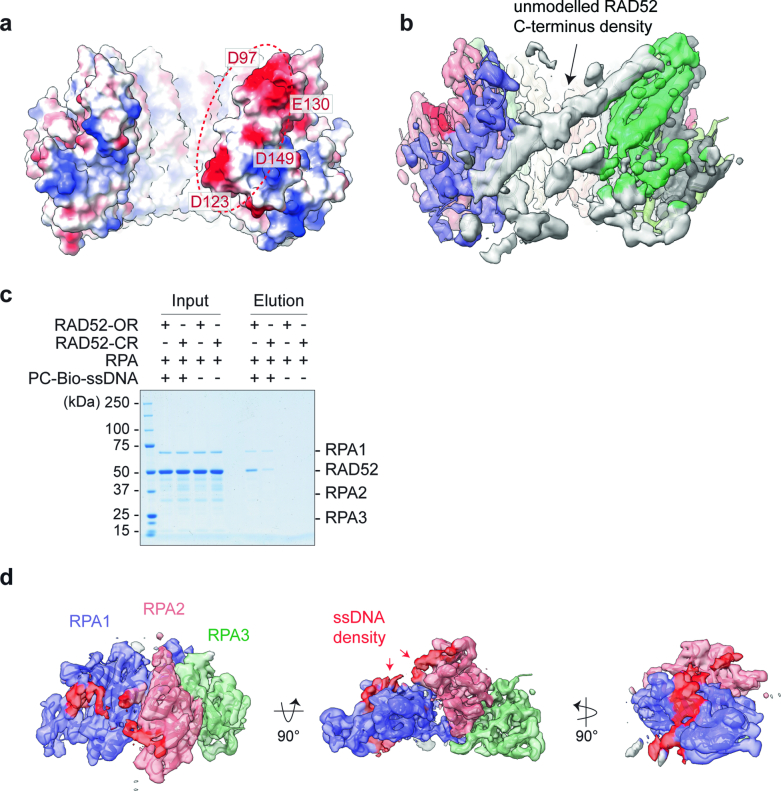
Structure of RPA-ssDNA and the RAD52-ssDNA-RPA complex. **a**, Surface view of a RAD52-ssDNA atomic model with electrostatic potential indicated (blue, positively charged; red, negatively charged). D97, D123, E130 and D149 are located at the opening of the RAD52 ring. **b**, Cryo-EM density of a RAD52-ssDNA sub-class separated by CryoSPARC 3D classification analysis with additional C-terminus density occupying the opening of the RAD52 ring. **c**, Coomassie blue stained SDS-PAGE of RAD52-ssDNA-RPA complex following streptavidin pull-down targeting biotin-labelled ssDNA with photocleavable linker (PC-Bio-ssDNA; 40 nt). **d**, Top, front and side view of the cryo-EM map of RPA-ssDNA complex (3.2 Å) with atomic model of the RPA trimeric core. ssDNA density (unmodelled) is coloured in red.

The RID and C-terminal sequences of RAD52 are highly conserved in vertebrates (Extended Data Fig. 6b). To determine whether these regions contribute to ring conformation, two deletion mutants, RAD52(∆RID) (deletion of amino acids 239–290) and RAD52(∆C) (deletion of amino acids 401–418), were generated (Extended Data Fig. 6c,d). An analysis of wild-type RAD52 using cation-exchange chromatography revealed a 2:1 ratio of open to closed rings, whereas RAD52(∆RID) and RAD52(∆C) exhibited an increased percentage of closed rings (Fig. 4c). Thermal melting analyses confirmed that the open and closed forms of the RAD52(∆RID) have a thermal stability similar to the wild-type RAD52, indicating that the mutant proteins have comparable open and closed architectures (Extended Data Fig. 6e). Given that the RID and the extreme C terminus are both positively charged (Extended Data Fig. 6f–h), whereas the N terminus of the exposed RAD52 subunit at site of ring opening is negatively charged (Extended Data Fig. 7a), these results indicate that these conserved regions interact through electrostatic interactions to prevent ring closure. In agreement, using focused 3D classification analysis, we found a subclass of RAD52–ssDNA particles that exhibited a low-resolution density across the site of ring opening corresponding to the C-terminal domain of RAD52 (Extended Data Fig. 7b).

## RAD52–RPA interactions are required for SSA

Two models of SSA can be considered (Fig. 4d): (1) annealing occurs by interactions between two RAD52–ssDNA complexes^20,37,38^; or (2) that a RAD52–ssDNA complex anneals with naked or RPA-coated ssDNA. To help to distinguish between these models, we analysed the efficiency of SSA at different concentrations of RAD52 and found that excess RAD52, sufficient to saturate both ssDNAs, inhibited single-strand annealing (Fig. 4e). Inhibition of SSA when the ssDNA is fully saturated by RAD52, and the requirement for RPA, leads us to suggest that model 2 should also be considered as a possible mechanism for SSA.

**Extended Data Fig. 8:**
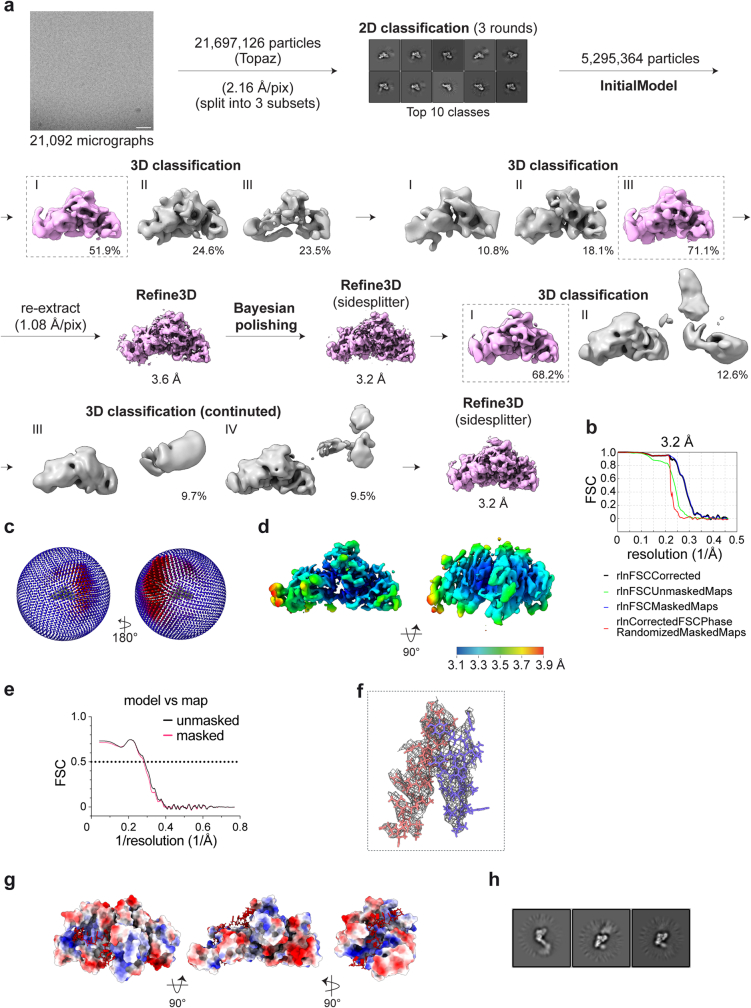
Cryo-EM data processing of RPA-ssDNA. **a**, As Extended Data Fig. 3 except for the RPA-ssDNA structure. **b**, Fourier Shell Correlation (FSC) plot. **c**, Angular distribution plot. **d**, Side and top views of RPA-ssDNA cryo-EM density coloured by local resolution. **e**, Model to map correlation graph. **f**, Zoom view of RPA-ssDNA cryo-EM map and atomic model focusing on 599-616aa of RPA1 (purple) and 152-174aa of RPA2 (pink). **g**, Top, front and side views of atomic model of RPA (trimeric core) presented as an electrostatic potential coloured surface. ssDNA model is present in stick style (red). **h**, Representative cryo-EM 2D class averages of RPA trimeric core with flexible density.

RAD52 open rings form more stable complexes with ssDNA (40 nucleotides) and RPA compared with RAD52 closed rings, as measured in pull-down experiments (Extended Data Fig. 7c), which may explain why the RAD52-CRs do not cooperate with RPA in SSA. To provide insights into the mechanism of annealing and to determine the role of RPA in the process, we next determined the cryo-EM structure of the RPA–ssDNA complex (3.2 Å) (Extended Data Figs. 7d and 8a–f). The density of the RPA trimeric core (47.9 kDa; RPA1 DBD-C, RPA2 DBD-D and RPA3 DBD-E) was well resolved, and we observed some additional density in RPA1 and RPA2 that corresponded to ssDNA. The molecular architecture of the human trimeric core was similar to its yeast and fungal orthologues^39,40^, with ssDNA bound to the positively charged groove across RPA1 DBD-C and RPA2 DBD-D (Extended Data Fig. 8g). Additional flexible densities, corresponding to DBD-A and DBD-B of RPA1 and the winged helix domain (WHD) (Extended Data Fig. 8h), were also observed, indicating that human RPA is structurally dynamic.

**Extended Data Fig. 9:**
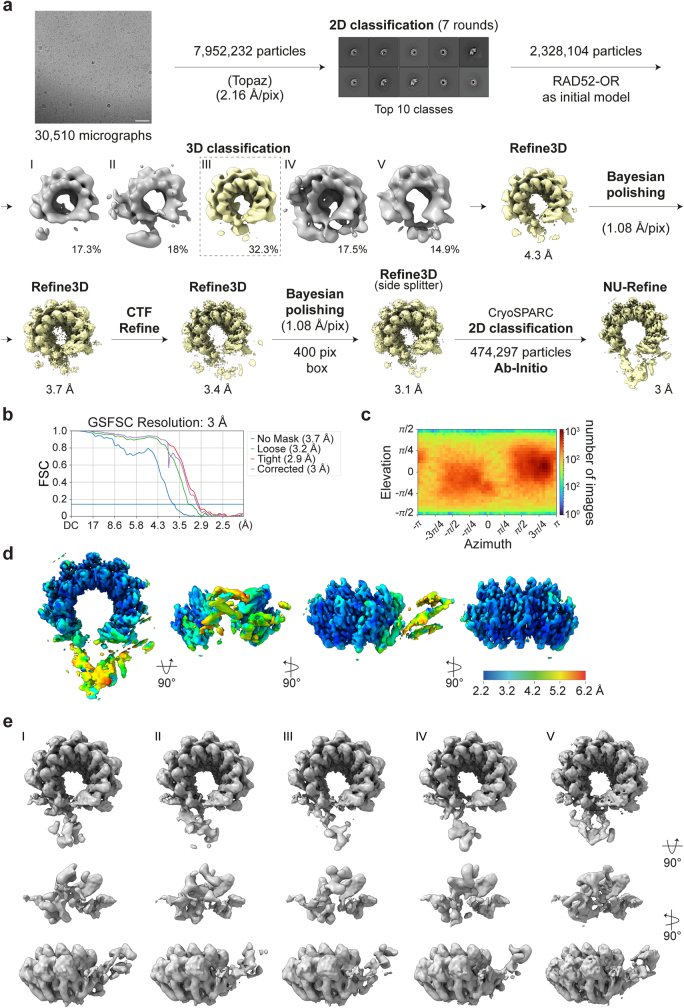
Cryo-EM data processing of RAD52-ssDNA-RPA complex. **a**, As Extended Data Fig. 3 except for the RAD52-OR-ssDNA-RPA structure. **b**, Fourier Shell Correlation (FSC) plot. **c**, Angular distribution plot. **d**, Top, front, side and back views of RAD52-ssDNA-RPA cryo-EM density coloured by local resolution estimated by CryoSPARC. **e**, Top, front and side views of 5 classes of the RAD52-ssDNA-RPA complex separated by focused 3D classification in CryoSPARC.

Analysis of the RAD52–ssDNA–RPA complex using negative-stain EM revealed no evidence of RAD52–RAD52 interactions, nor did we observe RPA bound to several RAD52 subunits around the open ring. Instead, we observed a single RPA protein sitting at the site of ring opening (Fig. 4f). These RAD52–ssDNA–RPA complexes were analysed using cryo-EM (3 Å), revealing that the RAD52 and ssDNA exhibited features similar to those present in the RAD52-OR–ssDNA cryo-EM structure (Fig. 4g and Extended Data Fig. 9a–d). Given that ssDNA was initially bound to RPA during sample preparation, these results would be consistent with the transfer of ssDNA from RPA to the RAD52 ring. The additional density corresponding to RPA at the opening of the RAD52 ring was not well resolved due to the inherent flexibility of the complex, and the local resolution around the RPA was low compared with RAD52–ssDNA (Extended Data Fig. 9d). CryoSPARC 3D classification analyses, focusing on the RPA, revealed various conformational states of the RPA density (Extended Data Fig. 9e) reflecting the heterogeneity and dynamics of the RAD52–RPA complex as observed by negative-stain EM (Fig. 4f).

## Interactions between the RID of RAD52 and RPA2

**Fig. 5:**
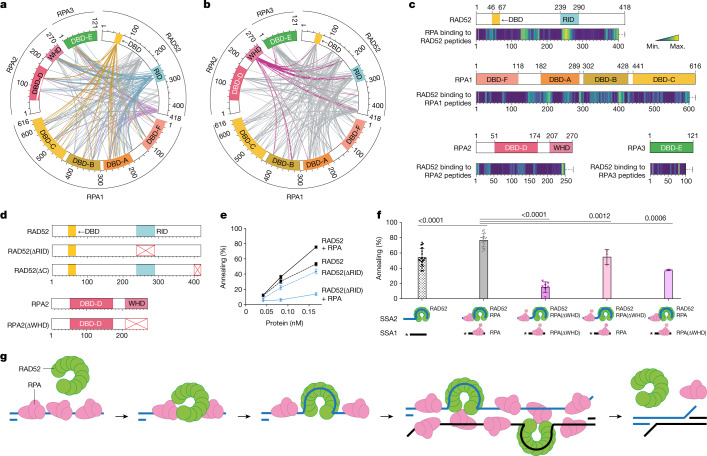
Interactions between the RID of RAD52 and the WHD of RPA2 are important for SSA. **a**,**b**, XL-MS analyses of the RAD52–ssDNA–RPA complex. The circos plots depict cross-links between RAD52-OR and RPA (RPA1, RPA2 and RPA3). Cross-links between the RAD52 DBD and RPA (yellow), the RAD52 RID and RPA (cyan), the extreme C terminus of RAD52 and RPA (light purple) and the WHD (dark pink) are highlighted. **c**, Peptide arrays of RAD52, RPA1, RPA2 and RPA3 showing interactions between RAD52 and RPA. The detected interaction intensities are shown as heat maps (yellow, maximum (max.) signal; purple, minimum (min.) signal). **d**, Schematic of RAD52, RAD52(∆RID), RAD52(∆C), RPA2 and RPA2(∆WHD). **e**, SSA mediated by the indicated proteins. Data are mean + s.e.m. *n* = 22 (RAD52 and RAD52 + RPA) and *n* = 3 (RAD52(∆RID) and RAD52(∆RID) + RPA) independent experiments. The ssDNA was 68 nucleotides. **f**, SSA catalysed by RAD52-OR and RPA or RPA(∆WHD), as indicated in the schematics (WHD deletion is indicated by a red cross). Data are mean ± s.e.m. The concentrations were as follows: ^32^P-labelled SSA1 and SSA2 (0.33 nM; 68 nucleotides), RPA (0.33 nM) and RAD52 (0.17 nM). All reactions were *n* = 3, except *n* = 22 (RAD52 and RAD52 + RPA) and *n* = 7 (RAD52 + RPA(∆WHD)), where *n* relates to independent experiments. Statistical analysis was performed using unpaired two-tailed *t*-tests. **g**, A model for SSA by interactions between RAD52 and RPA. First, RPA binds to and protects resected ssDNA. Second, RAD52 interacts with RPA-bound ssDNA, and ssDNA wraps around RAD52. The RAD52–ssDNA complex then interacts with RPA–ssDNA, leading to complementary-strand annealing. Finally, RAD52 and RPA dissociate from the annealed dsDNA.

**Extended Data Fig. 10:**
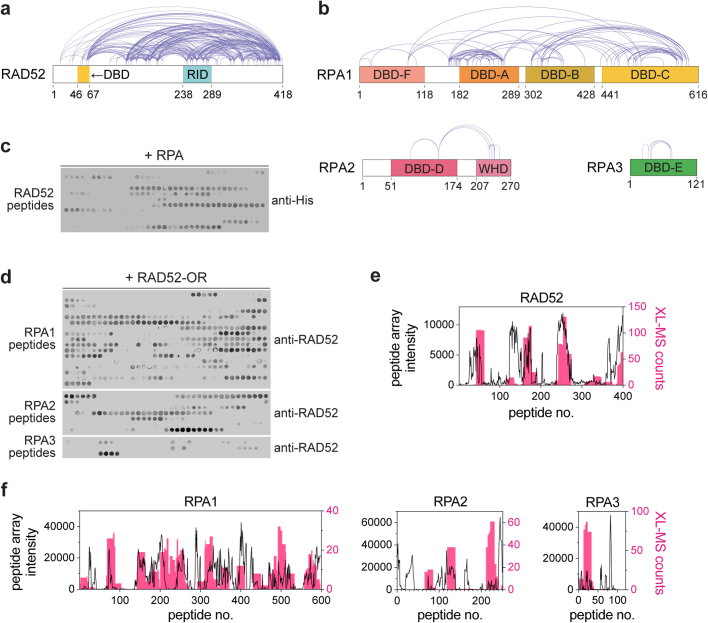
RAD52-RPA interactions. **a**, and **b**, Crosslinking mass spectrometry analysis of the RAD52-ssDNA-RPA complex. Diagram depicts crosslinks detected within RAD52-OR and RPA1, RPA2 or RPA3. **c**, and **d**, Peptide arrays of RAD52, RPA1, RPA2 and RPA3 showing interactions between RAD52 and RPA (related heat maps are shown in Extended Data Fig. 6c). Each spot corresponds to a 20 aa peptide sequence, and the intensity of signal reflects the strength of interaction between target proteins and peptides. **e** and **f**, Comparison of peptide arrays and XL-MS data for RAD52-RPA interactions. Crosslinked amino acid residues were transformed into counts per peptide corresponding to peptides used in the arrays.

XL-MS was then used to analyse critical interactions between RAD52 and RPA. In addition to the RID of RAD52 (amino acids 239–290)^21,33,37^, we found that the DBD (amino acids 46–67) and the extreme C terminus (amino acids 401–418) of RAD52 interacted with RPA (Fig. 5a,b and Extended Data Fig. 10a,b). These two domains are dynamically positioned at the opening of the RAD52 ring, which allows RPA to interact with RAD52 at the site of ring opening. Notably, the DBD of RAD52 interacted specifically with DBD-A, DBD-B and DBD-C of RPA1, DBD-D of RPA2 and DBD-E of RPA3. One possibility is that the interactions between DNA-binding sites in both proteins facilitate the hand-over of ssDNA from RPA to RAD52 at, or close to, the site of ring opening. As expected from previous studies^21,33,41^, we observed interactions between RPA1 and RAD52, as well as interactions between the WHD of RPA2 and RAD52. The XL-MS data were well supported by quantitative information of RAD52–RPA interactions derived from peptide array analyses (Fig. 5c and Extended Data Fig. 10c–f).

**Extended Data Fig. 11:**
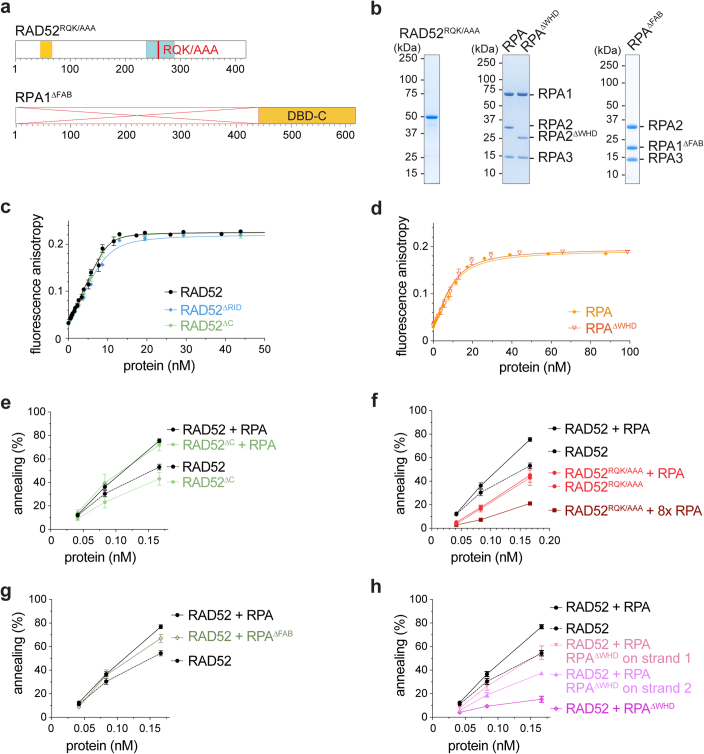
RAD52-RPA interactions involved in SSA. **a**, Schematic of RAD52^RQK/AAA^ (R260A, Q261A and K262A) and RPA1^∆FAB^ (∆2-440aa). **b**, SDS-PAGE of RAD52^RQK/AAA^, RPA, RPA^∆WHD^ and RPA^∆FAB^. **c**, Binding to ssDNA (10 nM, FAM-SSA4; 40 nt) by RAD52-OR, RAD52^∆RID^ and RAD52^∆C^ measured by fluorescence anisotropy, as in Fig. 1c. Equilibrium dissociation constants (*K*_D_) for ssDNA are 0.3 ± 0.1 nM (RAD52-OR), 0.7 ± 0.2 nM (RAD52^∆RID^-OR) and 0.2 ± 0.2 nM (RAD52^∆C^). Lines are best quadratic curve fits. Each point and error bar denotes mean + s.e.m. (RAD52-OR, n = 6; RAD52^∆RID^, n = 3; RAD52^∆C^, n = 3). n values are independent experiments. **d**, RPA and RPA^∆WHD^ binding to ssDNA (10 nM, FAM-SSA4; 40 nt) measured by fluorescence anisotropy. Equilibrium dissociation constants (*K*_D_) for ssDNA are 3.6 ± 0.5 nM (RPA) and 3.4 ± 0.9 nM (RPA^∆WHD^). Lines are best quadratic curve fits. Each point and error bar denotes mean + s.d. (RPA, n = 3; RPA^∆WHD^, n = 2). n values are independent experiments. **e**, SSA by RAD52-OR or RAD52^∆C^, with or without RPA, as in Fig. 1e. Each point and error bar denotes mean + s.e.m. (RAD52 and RAD52 + RPA, n = 22; RAD52^∆C^ and RAD52^∆C^ + RPA, n = 3). (ssDNA: 68 nt) **f**, SSA (68 nt) by RAD52-OR or RAD52^RQK/AAA^, with or without RPA. Each point and error bar denotes mean + s.e.m. (RAD52 and RAD52 + RPA, n = 22; RAD52^RQK/AAA^, n = 6 RAD52^RQK/AAA^ + RPA and RAD52^RQK/AAA^ + 8xRPA, n = 3). n values are independent experiments. **g**, SSA by RAD52-OR with RPA or RPA^∆FAB^ (ssDNA: 68 nt). Each point and error bar denotes mean + s.e.m. (n = 3; except RAD52 and RAD52 + RPA, n = 22). n values are independent experiments. **h**, SSA by RAD52-OR with RPA or RPA^∆WHD^, as in Fig. 5f (ssDNA: 68 nt). RAD52 was bound to strand 2. RPA was bound to both strands or replaced by RPA^∆WHD^ as indicated. Each point and error bar denotes mean + s.e.m. (n = 3; except RAD52 and RAD52 + RPA, n = 22, and RAD52 + RPA^∆^^WHD^, n = 7). n values are independent experiments.

To investigate the importance of these interaction hotspots, RAD52(∆RID), RAD52(RQK/AAA) (RAD52 mutated at the key residues R260A, Q261A and K262A in the RPA-interaction domain)^33,37^, RAD52(∆C), RPA(∆FAB) (deletion of DBD-F, DBD-A and DBD-B) and RPA(∆WHD) were purified (Fig. 5d and Extended Data Fig. 11a–d). We found that the RAD52(∆C) mutant exhibited a similar annealing activity to that of wild-type RAD52 in the presence or absence of RPA (Extended Data Fig. 11e). However, the presence of RPA inhibited SSA by RAD52(∆RID) (Fig. 5e). In contrast to the RID deletion, the RAD52(RQK/AAA) mutant was only inhibited when excess RPA was present (Extended Data Fig. 11f), indicating that other residues in the RID may also contribute to the interaction with RPA.

Analysis of the mutant RPAs showed that RPA(∆FAB) stimulated RAD52-mediated SSA (Extended Data Fig. 11g), whereas RPA(∆WHD) inhibited the reaction (Fig. 5f and Extended Data Fig. 11h). The inhibitory effect of RPA(∆WHD) was particularly evident when bound to the complementary strand (strand 2) that interacts with the initiating RAD52–ssDNA complex (strand 1) (Fig. 5f). These data show that the RPA-interacting domain of RAD52, together with the WHD of RPA2, have critical roles in mediating interactions between a RAD52–ssDNA complex and complementary RPA-coated ssDNA leading to ssDNA annealing.

In summary, we have obtained high-resolution structures of RAD52, RAD52–ssDNA complex, the trimeric core of RPA and, importantly, a RAD52–ssDNA–RPA complex that provide insights into the mechanism of annealing. We observed that ssDNA wraps around the RAD52 ring, along a positively charged groove and that nucleotide quartets adopt a length equivalent to B-form duplex DNA. At the conclusion of the annealing reaction, duplex DNA is released by RAD52.

Importantly, RAD52 adopts open- and closed-ring configurations, such that the open rings represent the active form of the protein for SSA. The closed rings may be an artifact of protein overexpression. Moreover, we observed that the C terminus of RAD52 contributes to the maintenance of the open-ring conformation, providing an explanation as to why the RAD52 NTD crystallized as closed rings^18,19^. Without separation of the open from closed rings, achieved here using cation-exchange chromatography, the heterogeneous nature of RAD52 complicates structural analysis and the interpretation of biochemical experiments^37,38^.

DNA annealing by the RAD52-ORs, but not RAD52-CRs, was stimulated by RPA, and involved specific interactions between the RPA-interacting domain of RAD52 and the WHD of RPA2. Notably, visualization of the RAD52–ssDNA–RPA complex revealed the presence of one RPA at the site of ring opening. Within this complex, the ssDNA-binding domain of RAD52 interacts specifically with DBD-A, DBD-B and DBD-C of RPA1, DBD-D of RPA2 and DBD-E of RPA3, indicating that these DNA-binding domains have an integrated role in the annealing reaction, possibly by mediating the hand-over of ssDNA from RPA to RAD52.

The observation that RPA stimulates RAD52-mediated SSA, and excess RAD52 inhibits the reaction, together with the lack of obvious RAD52–RAD52 interactions leads us to consider a new model for SSA. Initial events are likely to involve the sporadic binding of RAD52 to RPA-bound ssDNA, leading to the wrapping of ssDNA around the RAD52 ring. Then, rather than SSA being driven by interactions between two RAD52 rings^20,37,38^, in which the ssDNA is embedded within the DNA-binding grooves with consequential steric interactions that hinder rather than favour SSA, we suggest that SSA may involve direct interactions between RAD52–ssDNA and complementary ssDNA bound by RPA (Fig. 5g).

In human cells, RAD52 is expressed at low levels, and its concentration is around 5,000-fold lower than that of RPA^42^. Such a low level of expression would not be expected to support the displacement of RPA from ssDNA. Instead, we suggest that RPA has a series of direct roles in facilitating annealing: (1) RPA promotes the removal of secondary structures from ssDNA; (2) it mediates direct interactions with RAD52 and thereby targets RAD52 to the initiating ssDNA; and (3) the localization of RPA (together with the bound complementary strand) to the site of ring opening in RAD52 brings complementary sequences into close proximity such that annealing can occur on the surface of RAD52. The resulting annealed duplex DNA will then be released due to the low affinity that RAD52 exhibits for dsDNA.

The low expression levels of RAD52 may also limit SSA, which can lead to deletions between repeat sequences and loss of genetic information^43^. Low levels of expression will therefore favour non-mutagenic BRCA2/RAD51-mediated homologous recombinational repair. However, in human cancers, high expression levels of RAD52 have been observed and are associated with poor survival outcome^13,14^. Indeed, increased genome instability resulting from elevated levels of SSA may contribute to cancer cell growth and spread.

The synthetic lethal relationship between *RAD52* and *BRCA1/2* supports the notion that there may be therapeutic opportunities to specifically inhibit RAD52 in homologous-recombination-deficient cancer cells. With this in mind, several RAD52 inhibitors have been developed and show promising results in killing *BRCA2*-deficient cancer cells^15,16,44–48^. However, most inhibitors have a single mechanism of action in which they target the ssDNA binding activity of RAD52. The results presented here reveal the importance of the RAD52–RPA interaction in SSA and provide a potential future strategy for the specific inhibition of the annealing reaction by developing small-molecule inhibitors that interfere with RAD52–RPA interactions.

## Methods

### Purification of recombinant human RAD52

Human *RAD52* cDNA was codon optimized for expression in *E. coli* and cloned into pET100 (GeneArt, Thermo Fisher Scientific). Inverse PCR (primers: RAD52_tag_remove_F and RAD52_tag_remove_R) was performed to remove the 6×His, T7 and Xpress tags. The plasmid was transformed into BL21 Star (DE3) (Thermo Fisher Scientific) cells and a single colony was inoculated into an overnight culture using Luria broth (LB) supplemented with 0.8% glucose and 100 μg ml^−1^ ampicillin. An aliquot was diluted into 2 l of LB containing glucose and ampicillin, to an optical density at 600 nm (OD_600_) of 0.1, and incubated in an orbital shaker at 37 °C and 180 rpm. When the culture reached an OD_600_ of 0.8, IPTG (0.5 mM; Thermo Fisher Scientific) was added to induce RAD52 expression and incubation continued for a further 3 h. The culture was collected by centrifugation at 3,300*g* for 15 min, and the cell pellet was resuspended in 1 vol of PBS and centrifuged again. The pellet was then resuspended in lysis buffer (25 mM MES pH 6.5, 0.5 M NaCl, 10% glycerol and 1 mM EDTA) supplemented with Halt protease inhibitor (Thermo Fisher Scientific) and 0.25 mM TCEP, and lysed with Emulsiflex C5 (Avestin) at 4 °C. The lysate was clarified by centrifugation at 60,000*g* and 4 °C for 10 min. The supernatant was collected and diluted dropwise with the same lysis buffer without NaCl to reach 300 mM NaCl. The lysate was then clarified again by centrifugation at 60,000*g* and 4 °C for 20 min and loaded onto a HiTrap SP column (Cytiva) connected to an ÄKTA pure system at 4 °C. The column was washed with 3 column volumes (CV) of buffer containing 25 mM MES pH 6.5, 0.3 M NaCl, 1 mM EDTA, 10% glycerol and 0.25 mM TCEP, and eluted with 10 CV of a linear gradient of the same buffer containing 0.3–1 M NaCl. Peak fractions were diluted 3× with buffer containing 100 mM HEPES pH 7.0, 0.25 mM TCEP and Halt protease inhibitor and loaded onto a HiTrap Q column (Cytiva), that was eluted with 10 CV of a linear gradient (0.1–1 M NaCl) of HEPES buffer containing 0.5 mM EDTA and 0.25 mM TCEP. The HiTrap Q flow-through fraction was collected as crude purified RAD52.

To separate the two RAD52 conformations, RAD52 was loaded onto a Resource S column (Cytiva). Chromatography was performed using buffer containing 25 mM HEPES pH 7.0, 0.25 mM TCEP and various concentrations of NaCl. The Resource S column was (1) washed with 3 CV of 150 mM NaCl buffer; (2) eluted with 5 CV of linear gradient of 0.2–0.278 M NaCl buffer (until the conductivity was equivalent to 24.4 mS cm^−1^); (3) washed with 5 CV of 0.278 M NaCl buffer; and (4) eluted with 10 CV of 0.278–0.6 M NaCl buffer. The peak fractions of the two RAD52 forms were collected separately. RAD52-OR and RAD52-CR were loaded onto a Superose 6 Increase 10/300 GL column (Cytiva) using buffer containing 25 mM HEPES pH 8.0, 200 mM KOAc, 10% glycerol and 0.25 mM TCEP. The peak fractions were collected, aliquoted, snap-frozen in liquid nitrogen and stored at −80 °C. RAD52 concentrations were measured at a wavelength of 280 nm using the Nanodrop (Thermo Fisher Scientific) system and calculated as an 11-subunit ring (RAD52-CR and RAD52 NTD) or 10-subunit ring (RAD52-OR) with the exception that protomer concentration was used for circular dichroism (CD) analyses.

For the RAD52 NTD, inverse PCR (primers: RAD52_NTD_F and RAD52_NTD_R) was used to remove the C terminus (amino acids 210–418). The RAD52 NTD was purified using the same method as the full-length protein except that a linear gradient of 0.2–0.6 M NaCl was used for the Resource S column.

For RAD52(∆RID), RAD52(RQK/AAA) and RAD52(∆C), inverse PCR was used to remove the RPA-interacting domain (primers: RAD52_RID_F and RAD52_RID_R), extreme C terminus (primer: RAD52_NTD_F and RAD52_C_18D_R) and introduce the R260A, Q261A and K262A mutations (primer: RAD52_RQKAAA_F and RAD52_RQKAAA_R). All mutants were purified using the same method as for the full-length protein.

### Purification of Flag–RAD52 from Sf9 insect cells

Human *RAD52* cDNA was codon optimized for expression in Sf9 insect cells and cloned into pFastBac1 baculovirus expression vector with an N-terminal Flag tag (GeneArt, Thermo Fisher Scientific). The plasmid was transformed into DH10Bac (Thermo Fisher Scientific), and the bacmids were isolated with PureLink HiPure Plasmid Miniprep kit (Thermo Fisher Scientific). Overall, the generation and handling of the baculovirus was performed according to the Invitrogen Bac-to-Bac Baculovirus Expression System user manual with some modifications. In brief, recombinant bacmids were transfected into Sf9 cells with FuGENE HD, and P1 viruses were collected 66–72 h after transfection. The baculovirus titre was determined by isolating the viral DNA with High Pure Viral Nucleic Acid Kit (Roche), and quantitative PCR using Platinum qPCR supermix UDG (Thermo Fisher Scientific) and BaculoQUANT kit (Oxford Expression Technologies). The P2 baculovirus was amplified by infecting Sf9 cells at a multiplicity of infection (MOI) of 0.01 and 2 million cells per ml, and collected at 66–72 h after infection.

P2 baculovirus (MOI = 1) was used for recombinant Flag–RAD52 expression. Sf9 cells were grown in Sf-900 III SFM (Gibco, Thermo Fisher Scientific) at 27 °C in an orbital shaker at 140 rpm. The Sf9 cells were infected for 66–72 h. Cells were collected by centrifugation at 500*g* for 5 min and washed once with PBS. The cell pellet was resuspended in lysis buffer (25 mM MES pH 6.5, 600 mM NaCl, 10% glycerol and 1 mM EDTA) supplemented with Halt protease inhibitor (Thermo Fisher Scientific) and 0.25 mM TCEP, and sonicated in ice/water slurry at 25 amplitude for 150 s (with 1 s intervals to prevent warming) using a qSonica Q700 sonicator. The lysate was clarified by centrifugation at 60,000*g* for 30 min at 4 °C.

Pre-equilibrated anti-Flag M2 agarose beads (Merck) were added to the lysate, and the mixture was incubated on a rotator at 4 °C for 1.5 h. The beads were pelleted by centrifugation at 500*g* for 5 min at 4 °C and transferred to a gravity flow chromatography column. The column was washed extensively with the lysis buffer, and subsequently with buffer containing 25 mM HEPES pH 7.0, 450 mM NaCl, 10% glycerol, 1 mM EDTA, 0.25 mM TCEP and Halt protease inhibitor. The last wash was performed with the same buffer at 300 mM NaCl. Flag–RAD52 was then eluted with the buffer containing 450 mM NaCl and 0.5 mg ml^−1^ Flag peptide. Elution was performed twice by incubating the beads with an equal volume of elution buffer for 1 h at 4 °C. The eluates were combined, and diluted 4× using the same elution buffer at 100 mM NaCl without Flag peptide to lower the NaCl concentration to 150 mM. Resource S chromatography was performed as described above.

### Purification of recombinant human RPA

Human *RPA1*, *RPA2* and *10×His-RPA3* were synthesized and cloned into the pFastBac1 baculovirus expression vector (GeneArt, Thermo Fisher Scientific). *RPA1* (2 copies), *RPA2* and *10×His-RPA3*, together with their polyhedrin promoters, were then assembled into pBIG1a (biGBac multigene baculovirus expression vector)^49^ using Gibson assembly (NEB). Bacmids and baculovirus were generated as described above. P2 baculovirus (MOI = 1) was used for recombinant RPA expression. Sf9 cells were grown in Sf-900 III SFM (Gibco, Thermo Fisher Scientific) at 27 °C in an orbital shaker at 140 rpm, and infected for 66–72 h. Cells were collected by centrifugation at 500*g* for 5 min and washed once with PBS. The cell pellet was resuspended in buffer containing 25 mM HEPES pH 8.0, 0.5 M NaCl, 10% glycerol, 0.01% Tween-20, 20 mM imidazole, Halt protease inhibitor and 0.25 mM TCEP, and sonicated in ice/water slurry at 25 amplitude for 150 s (with 1 s interval to prevent warming) with a qSonica Q700 sonicator. The lysate was clarified by centrifugation at 60,000*g* for 30 min at 4 °C.

Pre-equilibrated Ni-NTA beads (Qiagen) were added to the lysate and the mixture was incubated on a rotator at 4 °C for 1 h. The beads were pelleted by centrifugation at 500*g* for 5 min at 4 °C and transferred to a chromatography column. The column was washed extensively with lysis buffer excluding Tween-20 while gradually decreasing the NaCl concentration from 0.5 to 0.2 M. Recombinant RPA was eluted with buffer containing 25 mM Tris-HCl pH 8.0, 0.2 M NaCl, 10% glycerol, 250 mM imidazole, Halt protease inhibitor and 0.25 mM TCEP. The RPA eluate was diluted 2× with the same elution buffer, without NaCl and imidazole, to lower the NaCl concentration to 100 mM. The diluted eluate was then loaded onto a Resource Q column (Cytiva) and eluted with linear gradient of buffer containing 0.1–0.4 M NaCl, 25 mM HEPES pH 8.0, 10% glycerol and 0.25 mM TCEP. Peak fractions containing RPA were loaded onto a Superdex 200 Increase 10/300 GL column (Cytiva) using buffer containing 25 mM HEPES pH 8.0, 200 mM KOAc, 0.5 mM EDTA, 10% glycerol and 0.25 mM TCEP. The protein was collected, aliquoted, snap-frozen in liquid nitrogen and stored at −80 °C.

For RPA1(∆FAB) and RPA2(∆WHD), inverse PCR was used to remove the DBD-F, DBD-A and DBD-B of RPA1 (amino acids 2–440) (primers: DBDC_F and DBDC_R) and the WHD of RPA2 (amino acids 207–270) (primers: RPA2_WHD_F and RPA2_WHD_R). Both deletion mutants were purified using the same method as described for the full-length protein.

### Oligonucleotides

All DNA oligonucleotides were HPLC purified (Merck and Integrated DNA Technologies). The names and sequences of the oligos were as follows where FAM is 6-carboxyfluoroscein: RAD52_tag_remove_F (5′-AGCGGCACCGAAGAAGCAATTTTAGG-3′), RAD52_tag_remove_R (5′-CATATGTATATCTCCTTCTTAAAGTTAAACAAAATTATTTCTAGAGGGG-3′), RAD52_NTD_F (5′-TAAAAGGGCGAGCTCAACGATCCGGCTG-3′), RAD52_NTD_R (5′-ACGACAGCTATTATAACGTGCTTCTTCAACGCTCGG-3′), RAD52_RID_F (5′-CCTCCGGCACCGCCTGTTAC-3′), RAD52_RID_R (5′-ATCCTGATCTGCCGGAATAACTGCATG-3′), RAD52_RQKAAA_F (5′-CGCACAGCTGCAACAGCAGTTTCGTGAACGTATGG-3′), RAD52_RQKAAA_R (5′-GCAGCCAGTTTACGCTGATGGGTTGCTTCGCTTTCAACTGCG-3′), RAD52_C_18D_R (5′-ATTACCGGTGGTACGCTGATCTGCGCTATAGG-3′), DBDC_F (5′-AACTGGAAAACCTTGTATGAGGTCAAATCCGAGAACCTGGG-3′), DBDC_R (5′-CATGGATCCGCGCCCGATGGTGG-3′), RPA2_WHD_F (5′-GCGGCCGCTTTCGAATCTAGAGCCTG-3′), RPA2_WHD_R (5′-AGTGAGGCCATTTGCTGGCATGAAGCTATTCC-3′), SSA1 (5′-TATCGAATCCGTCTAGTCAACGCTGCCGAATTCTACAGAGTTTGGGCTCCTCAACCTGCAGGTT-3′), SSA2 (5′-AACCTGCAGGTTGAGGAGCCCAAACCTCACTGGTAAATTCGCAGCGTTGACTAGACGGATTCGATA-3′), FAM-SSA4 (40nt) (5′-FAM-TATCGAATCCGTCTAGTCAACGCTGCCGAATTCTACCAGT-3′), SSA5 (5′-ACTGGTAGAATTCGGCAGCGTTGACTAGACGGATTCGATA-3′), SSA6 (5′-TGACCATCTTAAGCCGTCGCAACTGATCTGCCTAAGCTAT-3′), SSA7 (5′-CGGCAGCGTTGACTAGACGGATTCGATA-3′), gap 1-1 (5′-CGTGAAGTCGCCGACTGAATGCCAGCAATCTCTTTTTGAGTCTCATTTTGCATCTCGGCAATCTCTTTCTGATTGTCCAGTTGCATTTTAGTAAGCTCTTTTTGATTCTCAAATCCGGCG-3′), gap 1-2 (5′-CGCCGGATTTGAGAATCAAAAAGAGCTTAC-3′) and gap 1-3 (5′-GATTGCTGGCATTCAGTCGGCGACTTCACG-3′). Cy3- and Cy5-labelled and biotinylated oligonucleotides were purchased (Merck). To generate FAM-SSA1/SSA2 dsDNA, equimolar concentrations of FAM-SSA1 and SSA2 were mixed in 10 mM Tris-HCl pH 7.5, 100 mM NaCl and 1 mM EDTA, heated to 90 °C and gradually cooled to room temperature. Gapped DNA was annealed as described using gap 1-1, gap 1-2 and gap 1-3. Concentrations were measured using a spectrophotometer using absorbance values at 260 nm. All DNAs were stored at −20 °C.

### Fluorescence anisotropy

DNA-binding reactions (20 μl) were performed at 25 °C in buffer containing 25 mM HEPES pH 8.0, 0.2 M KOAc, 10% glycerol, 0.25 mM TCEP, 1 mM Mg(OAc)_2_ and 0.01% Brij-35. Proteins were serially diluted and mixed with 10 nM (final concentration) of FAM-labelled DNA in 384-well microplates (Corning). The plates were measured using the CLARIOstar microplate reader (BMG Labtech). Blank-corrected anisotropy measurements were averaged and plotted against protein concentration. RAD52 binding was curve-fitted using the following quadratic equation in GraphPad Prism 9 to determine *K*_D_ values:

$$Y={A}_{\min }+\left({A}_{\max }{-A}_{\min }\right)\times \frac{x+L+{K}_{{\rm{D}}}-\sqrt{{\left(x+L+{K}_{{\rm{D}}}\right)}^{2}-4\times x\times L}\,}{2\times L},$$

where *Y* is the fluorescence anisotropy, *A*_min_ and *A*_max_ are the minimum and maximum fluorescence anisotropy values, *L* is the ligand concentration (equal to 0.01 µM), *x* is the protein concentration and *K*_D_ is the dissociation constant. At least three independent triplicates of technical replicates were performed for each binding condition.

### Single-stranded DNA annealing

Reactions (15 μl) contained 5′-^32^P-labelled SSA1 (68 nucleotides) with its complementary strand SSA2 (68 nucleotides)^30^ in 25 mM HEPES pH 8.0, 0.2 M KOAc, 1 mM Mg(OAc)_2_, 0.01% Brij-35, 0.25 mM TCEP and 5% glycerol. Two separate 7.5 µl reaction mixtures were set up. One contained 5′-^32^P-labelled SSA1 (0.33 nM) in buffer, and the second contained SSA2 (0.33 nM). RPA (0.33 nM) was added to both, as indicated. RAD52 (0.33 nM, or as indicated in figure legends) was added to SSA2 and incubated for 10 min at 25 °C. The two tubes were then mixed and incubated for 10 min at 25 °C, before being stopped by deproteinization using 3 µl of proteinase K (20 mg ml^−1^ proteinase K in 10 mM Tris-HCl pH 7.5 and 1 mM CaCl_2_) and incubated at 30 °C for 30 min. The samples were supplemented with Ficoll loading buffer and analysed by PAGE with TBE as the running buffer. Gels were dried and exposed to phosphorimaging plates and images acquired using the Typhoon FLA 9500 biomolecular imager (GE) and quantified using ImageJ^50,51^.

For reactions using 40-nucleotide ssDNA (5′-^32^P-labelled SSA4 with complimentary SSA5), the reactions were set up as described above except that the concentration of ssDNA was lowered to 0.13 nM to prevent self-annealing of ssDNA, and 0.13 nM of RPA was used. Concentrations of RAD52 are indicated in figure legends.

To determine whether DNA ends were required for RAD52-OR mediated annealing, interactions between 0.33 nM circular φX174 virion ssDNA and 0.33 nM ^32^P-labelled gapped duplex DNA (a 60-nucleotide-long ssDNA that had 30-mers annealed to each end) were analysed. For these experiments, RPA (0.33 nM or 19.9 nM) was premixed with the gapped and circular ssDNAs, respectively (to provide similar coverage). RAD52 was then added to the gapped ssDNA and annealing was measured by electrophoresis through a 1% agarose gel using TAE buffer.

To analyse ssDNA annealing using size-exclusion chromatography, RAD52-OR (4 µM) was preloaded on SSA2–Cy5 (4 µM, 12.5 µl) before an equal volume of Cy3–SSA1 (4 µM) was added. After 30 min on ice, the reaction was loaded onto the Superdex 200 Increase 3.2/300 column connected to the ÄKTA pure Micro system. Chromatography was performed at 4 °C with buffer containing 25 mM HEPES pH 8.0, 200 mM KOAc, 0.25 mM TCEP and 1 mM Mg(OAc)_2_.

### Biolayer interferometry analysis

40-nucleotide (SSA4) ssDNA was biotinylated at either the 5′ or 3′ end (indicated as bio–ssDNA or ssDNA–bio, respectively). 68-nucleotide (SSA1) ssDNA was biotinylated at the 3′ end (indicated as SSA1–bio), and 28 nucleotides of complementary ssDNA was annealed to the 5′ end to protect the 5′ ssDNA end (indicated as ds-ssDNA–bio). The experiments were performed using the Octet R8 system (Sartorius) at 25 °C in buffer containing 25 mM HEPES pH 8.0, 200 mM KOAc, 0.01% Tween-20, 1 mM Mg(OAc)_2_ and 0.25 mM TCEP. The biotinylated DNA substrates (5 nM) were immobilized onto Octet SA streptavidin biosensors until a 0.05 threshold, and the sensors were then moved to wells containing a range of RAD52 concentrations (20, 10, 5, 2.5, 1.25, 0.625 and 0.312 nM). The association of RAD52 to DNA was recorded for 60 min and the dissociation for 5 min using the Octet BLI Discovery Software. Equilibrium dissociation constants (*K*_D_) were obtained by plotting association amplitudes at equilibrium versus protein concentration (Octet Analysis Studio Software; Sartorius) and plotted in GraphPad Prism 9. The following 1:1 binding equation was used to determine *K*_D_ values: using the following quadratic equation in GraphPad Prism 9 to determine *K*_D_ values:

$$Y={B}_{\max }\times X/({K}_{D}+X),$$

where *Y* is the association amplitude, *B*_max_ is the maximum amplitude at saturation, *X* is the protein concentration and *K*_D_ is the dissociation constant. Three independent triplicates were performed for each binding condition.

### CD analysis

Far-UV CD measurements were performed on a Jasco J-815 spectropolarimeter fitted with a cell holder temperature-regulated by a CDF-426S Peltier unit. Spectra were recorded at 20 °C at protein concentrations of 3.3 µM (RAD52-OR) and 3.2 µM (RAD52-CR) in 10 mM potassium phosphate buffer pH 8.0, 100 mM NaF and 0.25 mM TCEP. Fused silica cuvettes were used with a 1 mm path length (Hellma). Spectra were recorded at a resolution of 0.2 nm and were baseline corrected by subtraction of the appropriate buffer spectrum. CD intensities are presented as the molar CD extinction coefficient (∆*ε*_M_) calculated as:

$${\Delta \varepsilon }_{{\rm{M}}}=\frac{S}{\mathrm{32,980}\times {c}_{{\rm{M}}}\times L}\left({\rm{units:}}{{\rm{M}}}^{-1}{{\rm{cm}}}^{-1}\right),$$

where *S* is the signal in millidegrees, *c*_M_ is the molar concentration and *L* is the path length (in cm). Secondary structure content was estimated as described^52^.

### Intact protein MS

Proteins were diluted to 1 µM with 0.1% (v/v) formic acid and injected onto a C4 BEH 1.7 µm, 1.0 × 100 mm, UPLC column using the Acquity I class LC (Waters) system. Proteins were eluted with a 15 min gradient of acetonitrile (2% (v/v) to 80% (v/v)) in 0.1% (v/v) formic acid using a flow rate of 50 µl min^−1^. The analytical column outlet was directly interfaced through an electrospray ionization source, with a time-of-flight (TOF) mass spectrometer (BioAccord, Waters). Data were acquired over a *m*/*z* range of 300–8,000, in positive-ion mode with a cone voltage of 40 V. Scans were summed together manually and deconvoluted using MaxEnt1 (Masslynx, Waters). The parameters used were as follows; input *m*/*z* range (Da): 600–2,000; output mass range (Da): 30000–60000; TOF resolution: 10000.00; and iterate to convergence.

### GuHCl denaturation and renaturation

RAD52 (purified to the HiTrap Q step) was dialysed into 25 mM HEPES pH 7.0, 6 M GuHCl, 0.5 mM EDTA and 2 mM β-mercaptoethanol overnight at 4 °C. The denatured protein was analysed using a Superose 6 Increase 10/300 GL column, which was run with 6 M GuHCl buffer. Protein was renatured by dialysis in native buffer (25 mM HEPES pH 7.0, 200 mM NaCl, 0.5 mM EDTA and 2 mM mercaptoethanol) for 24 h at 4 °C. The renatured RAD52 was then run on the same column using native buffer. To analyse the percentage of open and closed rings, the renatured RAD52 sample was loaded onto the Resource S column.

### Negative-stain EM sample preparation and data acquisition

Samples (4 µl, 25 ng µl^−1^) were applied for 1 min to glow discharged (25 mA, 30 s) 400-mesh carbon-coated copper grids (C400Cu100, EM Resolutions). The grids were sequentially stained in four separate 30 µl droplets of 2% (v/v) uranyl acetate for 10, 15, 20 and 25 s. Excess uranyl acetate was blotted away from the grid using Whatmann paper, allowed to air dry and stored before imaging.

The grids were imaged on the Tecnai LaB_6_ G2 Spirit TEM operating at 120 kV equipped with a 2K Gatan Ultrascan 1000 camera. Micrographs were acquired manually using DigitalMicrograph at a nominal magnification of ×30,000 (3.5 Å per pixel) or ×42,000 (2.4 Å per pixel) with defocus values ranging from −0.7 to −1.5 µm.

### Negative-stain EM data analysis

DM3 files were converted to MRC format using e2proc2d.py (EMAN2)^53^. Micrographs were imported into Relion 3.1 or 4.1^54,55^, CTF parameters were calculated using CTFFIND4^56^, and particles were picked using crYOLO^57^ or Topaz^58^. Particles were extracted and iteratively 2D classified (ignore CTF to first peak = yes, limit resolution E-step = 20 Å, additional arguments = --only-flip-phases).

### Cryo-EM sample preparation

Recombinant RAD52 and RPA were purified to the Resource S or Resource Q step, and freshly purified on the Superose 6 Increase 10/300 GL or Superdex 200 Increase 10/300 GL column before making the cryo-EM grids. For RAD52-CR, the protein was in a buffer containing 25 mM HEPES pH 7.0, 150 mM NaCl and 0.25 mM TCEP, diluted to 0.3 mg ml^−1^, and supplemented with 0.00005% Tween-20. A sample (4 μl) was applied to freshly glow-discharged (45 mA, 60 s; Quorum Emitech K100X) Quantifoil R2/1 300 mesh copper grids and vitrified using a Vitrobot Mark IV (Thermo Fisher Scientific) cooled to 4 °C with 95% humidity. Grids were double-side blotted for 0.5 s and plunge frozen in liquid ethane. For RAD52-OR, the grids were prepared as described above except Quantifoil R2/2 200 mesh copper grids were used, and the concentration was 0.25 mg ml^−1^, the Tween-20 concentration was 0.001%, and blot time was 1.5 s. For RAD52-OR–ssDNA, the protein (0.25 mM) was diluted to 0.5 µM in 25 mM HEPES pH 8.0, 150 mM NaCl, 2 mM Mg(OAc)_2_ and supplemented with 0.05% octyl-β-glucoside (OG). SSA4 (1 µM) was added and incubated at 25 °C for 10 min. The concentration was determined by Bradford assay (Bio-Rad) and diluted to 0.15 mg ml^−1^ with the same buffer. Grids were prepared as above except Quantifoil R1.2/1.3 300 mesh copper grids were used and the blot time was 2.5 s. For RPA–ssDNA, the protein (0.25 mM), in 25 mM HEPES pH 8.0, 150 mM NaCl, 2 mM Mg(OAc)_2_, was diluted to 3 µM, and supplemented with 0.1 mM CHAPSO. SSA7 (6 µM) was added and incubated at 25 °C for 10 min. The concentration was determined using the Bradford assay (Bio-Rad) and diluted to 0.15 mg ml^−1^ with the same buffer. UltrAuFoil R2/2 200 mesh gold grids (Quantifoil) were prepared as described above and the blot time was 2.5 s. The RAD52-OR–ssDNA–RPA ternary complex was assembled as indicated in the ‘Reconstitution of the RAD52–ssDNA–RPA complex’ section below. The concentration was determined using the Bradford assay (Bio-Rad) and diluted to 0.1 mg ml^−1^ with buffer supplemented with 0.00075% Tween-20 and 0.075 mM CHAPSO. Quantifoil R2/2 200 mesh copper grids were prepared as described above, except the blot time was 3 s.

### Cryo-EM data collection, image processing and atomic model building

RAD52-CR and RAD52-OR datasets were collected on a Titan Krios Cryo-TEM equipped with a Falcon III direct electron detector (Thermo Fisher Scientific) at the Francis Crick Institute Structural Biology STP. The RAD52-OR–ssDNA dataset was collected on a Titan Krios G3i (FEI, Thermo Fisher Scientific) equipped with a Gatan K3 direct electron detector at the London consortium for cryo-EM (LonCEM). RPA–ssDNA and RAD52-OR–ssDNA–RPA datasets were collected on a Titan Krios Cryo-TEM (Thermo Fisher Scientific) equipped with a K2 direct electron detector (Gatan) at the Francis Crick Institute Structural Biology STP.

Single-particle analyses were performed within Relion (v.4.0)^54^ and CryoSPARC^59^. The videos were corrected for drift and dose-weighted using RELION’s own implementation of MOTIONCOR2^60^ and subsequent contrast transfer (CTF) parameters were measured using CTFFIND4^56^. Particles were picked automatically using crYOLO^57^ or Topaz^58^. Details of image processing are illustrated in Extended Data Figs. 3, 4, 5, 8 and 9. In brief, several rounds of 2D classification were performed to remove particles that cannot be aligned to yield defined 2D averages. Several rounds of 3D classifications were performed to separate different conformations or particles that cannot be aligned to yield high-resolution 3D volumes. 3D auto-refine, Bayesian polishing (minimum two rounds) and CTF refinement (minimum one round) were performed iteratively to achieve high resolution 3D reconstruction in RELION^61,62^. Polished particles were imported to CryoSPARC^59^, and refined using non-uniform refinement^63^. 3D variability^64^ or 3D classifications were performed to detect heterogeneity within the cryo-EM densities. The cryo-EM maps were sharpened by post-processing in RELION, CryoSPARC or DeepEMhancer^65^ if there was high variability in local resolution. The overall resolution is reported at FSC = 0.143 (ref. ^66^).

All model building was performed using Phenix^67,68^, COOT^69^ and ISOLDE^70^ in ChimeraX^71^. For RAD52-CR, the crystal structure of the RAD52 NTD (PDB: 1H2I) was placed into a sharpened RAD52-CR cryo-EM map in ChimeraX^71^ and initially refined using Namdinator^72^. One RAD52 subunit was removed from RAD52-CR and used for initial refinement in Namdinator for RAD52-OR. ssDNA was built manually in COOT into the RAD52-OR model using RAD52-OR–ssDNA as a starting model. RPA1, RPA2 and RPA3 AlphaFold2 models were used for Dock and rebuild in Phenix^73,74^ and the ssDNA model was aligned and extracted from the fungal RPA structure (PDB: 4GOP)^39^. The RAD52-OR–ssDNA model was used as the initial model for RAD52-OR–ssDNA–RPA.

### SEC–MALLS analysis

SEC–MALLS was used to determine the molar mass composition of RAD52. Purified RAD52-OR (2.0, 1.0 or 0.5 mg ml^−1^) was loaded onto a Superose 6 Increase 10/300 GL column connected to a Jasco chromatography system. Chromatography was performed at 25 °C with buffer containing 25 mM HEPES pH 7.0, 150 mM NaCl, 0.25 mM TCEP and 3 mM NaN_3_ at a flow rate of 1.0 ml min^−1^. RAD52-OR–ssDNA (2 mg ml^−1^) was analysed in a similar manner using 25 mM Bis-Tris propane pH 8.5, 200 mM NaCl, 5 mM MgCl_2_, 0.25 mM TCEP and 3 mM NaN_3_ as the running buffer. The scattered light intensity and protein concentrations of the column eluates were recorded using a DAWN-HELEOS laser photometer and an OPTILAB-rEX differential refractometer (d*n*/d*c* = 0.186). The weight-averaged molecular mass of material contained in chromatographic peaks was determined using the combined data from both detectors in the ASTRA software v.7.3.2 (Wyatt Technology).

### Nuclear/chromatin extraction and analysis

U2OS cells (authenticated and microplasma free, as determined by the Francis Crick Institute) were grown in DMEM (Gibco) supplemented with 10% FBS (Gibco) in humidified incubators at 37 °C and 5% CO_2_. Cells were collected from four confluent 500 cm^2^ square dishes and washed once with PBS. The pellet was supplemented with 5× pellet volume of CSK buffer (10 mM PIPES pH 6.8, 100 mM NaCl, 3 mM MgCl_2_, 300 mM sucrose, 1 mM EGTA, 0.5% Triton X-100 and 0.25 mM TCEP) supplemented with Halt protease and phosphatase inhibitors, incubated on ice for 10 min and centrifuged at 2,000*g* for 5 min at 4 °C. The supernatant was collected as the first CSK extract. A 3× pellet volume of CSK buffer (containing 0.1% Triton X-100) was added to the pellet, incubated on ice for 10 min and the sample was centrifuged at 3,000*g* for 5 min at 4 °C. The supernatant was collected as the second CSK extract. An equal volume of benzonase digestion buffer (20 mM HEPES pH 8.0, 2 mM MgCl_2_, 0.5% Triton X-100, 0.25 mM TCEP and 500 units benzonase/100 µl of buffer) supplemented with Halt protease and phosphatase inhibitors was added to the pellet and incubated on ice for 10 min. A 2× sample volume of high-salt buffer (20 mM HEPES pH 8.0, 600 mM NaCl and 0.25 mM TCEP) supplemented with Halt protease and phosphatase inhibitors was then added, incubated on ice for 10 min, and the sample was centrifuged at 21,000*g* for 10 min at 4 °C. The supernatant was collected as a nuclear/chromatin extract.

Glycerol gradients (5 ml, 10–30%) in 25 mM HEPES pH 8.0, 150 mM NaCl, 10–30% glycerol and 0.25 mM TCEP were cast in thin-wall polypropylene tubes (Beckman Coulter) using a Gradient Master (Biocomp) and kept in the cold room overnight to equilibrate to 4 °C. U2OS nuclear/chromatin extracts (200 µl), 200 ng recombinant RAD52-OR or a gel-filtration calibration marker (Cytiva) was loaded gently onto the top of three gradients, which were then centrifuged at 4 °C and 55,000 rpm (368,000*g*) using SW 55 Ti rotor (Beckman Coulter) for 4 h. The fractions were collected by manual pipetting from the top of the gradients. The U2OS nuclear/chromatin extract (500 µl), 500 ng recombinant RAD52-OR or a gel-filtration calibration marker (Cytiva) were also loaded onto the pre-equilibrated Superose 6 Increase 10/300 GL column (Cytiva). Chromatography was performed with a buffer containing 25 mM HEPES pH 8.0, 150 mM NaCl, 10% glycerol and 0.25 mM TCEP at 4 °C. Fractions were collected and analysed by SDS–PAGE followed by western blotting using antibodies against RAD52 (rabbit monoclonal, 1:500, Abcam, ab124971). Alexa Fluor Plus 800 anti-rabbit secondary antibodies (1:2,000, Invitrogen, A32735) were used and the membranes were imaged using an Odyssey DLx instrument with ImageStudio software (Licor).

### RAD52 Resource S chromatogram peak fitting

Resource S chromatography was performed as described above except a linear gradient of 0.2–0.6 M NaCl was used. The UV_280_ absorbance values were imported into GraphPad Prism 9 and curved fitted using a sum of two Gaussians equation to deconvolute open- and closed-ring peaks:

$$Y={\rm{amplitude}}\times \exp \left(-0.5\times {\left(\frac{X-{\rm{mean}}}{{\rm{s.d.}}}\right)}^{2}\right)+\mathrm{amplitude\; 2}\times \exp \left(-0.5{\left(\frac{X-\mathrm{mean\; 2}}{\mathrm{s.d.\; 2}}\right)}^{2}\right)$$

### RAD52–ssDNA–RPA pull downs

The RAD52–ssDNA–RPA ternary complex (400 μl) was reconstituted in buffer containing 25 mM HEPES pH 8.0, 200 mM KOAc, 2 mM Mg(OAc)_2_, 0.01% Tween-20 and 0.25 mM TCEP. Biotin-labelled SSA4 (0.1 μM), with photo-cleavable linker (Integrated DNA Technologies), and recombinant RPA (0.15 μM) were mixed and incubated on ice for 10 min. RAD52-OR (0.15 μM) was then added and incubation continued for a further 10 min. Pre-washed Streptavidin Sepharose Mag beads (10 μl, Cytiva) were then added and incubated for 30 min on a head-to-toe rotator at 4 °C. The beads were washed once with reaction buffer and then with reaction buffer Tween-20. The beads were resuspended in 20 μl reaction buffer, and irradiated with 365 nm UVA on ice/water slurry to cleave the photo-cleavable linker.

### Reconstitution of the RAD52–ssDNA–RPA complex

RAD52-OR (purified to the Resource S step) and RPA (purified to the Resource Q step) were loaded onto the Superose 6 Increase 10/300 GL (Cytiva) and Superdex 200 Increase 10/300 GL (Cytiva) columns, respectively, and run with buffer containing 25 mM HEPES pH 8.0, 150 mM NaCl, 2 mM Mg(OAc)_2_ and 0.25 mM TCEP. The reconstitution mixture for cryo-EM was supplemented with 0.00075% Tween-20 and 0.075 mM CHAPSO, whereas the XL-MS sample was supplemented with 0.05% OG. Reconstitution of the RAD52-OR–ssDNA–RPA ternary complex involved two steps: (1) RPA (1 µM final concentration) was added to SSA1 (0.5 µM final concentration) and incubated at 25 °C for 10 min; and (2) RAD52-OR (0.5 µM final concentration) was added and incubated at 25 °C for 30 min. The sample was centrifugated at 21,000*g* for 1 min at 4 °C before proceeding with cryo-EM grid preparation and XL-MS.

### Protein disorder prediction

The human RAD52 protein sequence (UniProt: P43351) was uploaded to the ODiNPred^75^ webserver (https://st-protein.chem.au.dk/odinpred). The predicted disorder probability of each residue was plotted in GraphPad Prism 9.

### Multiple-sequence alignment

RAD52 protein sequences from different organisms were aligned with Clustal Omega using the default settings^76^. The alignment was formatted with ESPript3.0^77^.

### XL-MS analysis

RAD52-OR and RAD52-OR–ssDNA–RPA ternary complexes (0.5 µM, reconstituted as above) were supplemented with a 1:100 molar ratio of disuccinimidyl dibutyric urea (DSBU: 50 µM) for 1 h at room temperature, before the mixture was quenched by the addition of NH_4_HCO_3_ to a final concentration of 20 mM (15 min at room temperature). The cross-linked proteins were reduced with 10 mM dithiothreitol and alkylated with 50 mM iodoacetamide. They were then digested with trypsin at an enzyme-to-substrate ratio of 1:100, for 1 h at room temperature and further digested overnight at 37 °C after addition of trypsin at a ratio of 1:20. The peptide digests were then fractionated batch-wise by high pH reverse-phase chromatography on micro spin TARGA C18 columns (Nest Group) into four fractions (10 mM NH_4_HCO_3_/10% (v/v) acetonitrile pH 8.0; 10 mM NH_4_HCO_3_/20% (v/v) acetonitrile pH 8.0; 10 mM NH_4_HCO_3_/40% (v/v) acetonitrile pH 8.0; and 10 mM NH_4_HCO_3_/80% (v/v) acetonitrile pH 8.0). The fractions (150 µl) were evaporated to dryness in a CentriVap concentrator (Labconco) before analysis by LC–MS/MS.

Lyophilized peptides were resuspended in 1% (v/v) formic acid and 2% (v/v) acetonitrile and analysed by nano-scale capillary LC-MS/MS using a Vanquish Neo UPLC (Thermo Fisher Scientific, Dionex) to deliver a flow of approximately 300 nl min^−1^. A PepMap Neo C18 5 μm, 300 μm × 5 mm nanoViper (Thermo Fisher Scientific, Dionex) trapped the peptides before separation on a 25 cm EASY‐Spray column (25 cm × 75 µm inner diameter, PepMap C18, 2 µm particles, 100 Å pore size, Thermo Fisher Scientific). Peptides were eluted with a gradient of acetonitrile. The analytical column outlet was directly interfaced through a nano-flow electrospray ionization source, with a quadrupole Orbitrap mass spectrometer (Orbitrap Exploris 480, Thermo Fisher Scientific). MS data were acquired in data-dependent mode using a top ten method, where ions with a precursor charge state of 1+ and 2+ were excluded. High-resolution full scans (*R* = 60,000, *m*/*z* 380–1,800) were recorded in the Orbitrap followed by higher-energy collision dissociation (HCD) (stepped collision energy 30 and 32% normalized collision energy) of the ten most intense MS peaks. The fragment ion spectra were acquired at a resolution of 30,000 and a dynamic exclusion window of 20 s was applied.

For data analysis, Xcalibur raw files were converted into the MGF format using Proteome Discoverer v.2.3 (Thermo Fisher Scientific) and used directly as input files for MeroX^78^. Searches were performed against an ad hoc protein database containing the sequences of the proteins in the complex and a set of randomized decoy sequences generated by the software. The following parameters were set for the searches: maximum number of missed cleavages: 3; targeted residues K, S, Y and T; minimum peptide length 5 amino acids; variable modifications: carbamidomethylation of cysteine (mass shift 57.02146 Da), methionine oxidation (mass shift 15.99491 Da); DSBU modified fragments: 85.05276 Da and 111.03203 Da (precision: 5 ppm MS and 10 ppm MS/MS); false-discovery-rate cut-off: 5%. Finally, each fragmentation spectrum was manually inspected and validated.

To compare with the peptide array experiments, the number of cross-links detected for each amino acid residue was counted, and summed within an individual 20 amino acid peptide with a 1 amino acid shift, similar to the peptide array. The overlayered result was plotted using GraphPad Prism 9.

### Peptide array

Peptides (20 amino acids) with 1-amino-acid shift covering the full sequences of RAD52, RPA1, RPA2 and RPA3 were synthesized on cellulose membranes in 3 mm spots by the Chemical Biology STP at the Francis Crick Institute. The membranes were washed with 50% ethanol and 10% acetic acid for 30 min and equilibrated with 1× TBST (50 mM Tris-HCl pH 7.5, 150 mM NaCl and 0.1% Tween-20) supplemented with 0.25 mM TCEP. The membrane was blocked with 5% non-fat milk in TBST (0.1% Tween-20) supplemented with 0.25 mM TCEP for 1 h at room temperature. To allow protein-peptide interactions, the membranes were incubated with RAD52-OR or RPA (1 µg ml^−1^) in 1% non-fat milk in TBST (0.1% Tween-20) supplemented with 0.25 mM TCEP overnight at 4 °C. The membranes were washed in 1× TBST (0.1% Tween-20) supplemented with 0.25 mM TCEP on an orbital shaker for 5 min at room temperature three times. The membranes were then incubated in primary antibodies (anti-His 1:1,000, Takara, 631212) in 1% non-fat milk in TBST (0.1% Tween-20) supplemented with 0.25 mM TCEP for 2 h at room temperature. The membranes were washed three times as before and incubated in Alexa-Fluor-Plus-conjugated secondary antibodies (goat anti-mouse 1:2,000, Thermo Fisher Scientific, A32730; goat anti-rabbit, 1:2,000, Thermo Fisher Scientific, A32735) in 1% non-fat milk in TBST (0.1% Tween-20) supplemented with 0.25 mM TCEP for 1 h at room temperature. The membranes were washed three times, imaged on a Li-Cor Odyssey DLx system and quantified using Image Studio Lite (Li-Cor).

### Nanoscale differential scanning fluorometry

A Prometheus NT-48 (Nanotemper) instrument was used to monitor changes in tryptophan fluorescence following thermal denaturation. Proteins were diluted to 10 µM in 25 mM HEPES pH 8.0, 200 mM KOAc, 0.5 mM EDTA, 10% glycerol and 0.25 mM TCEP. The samples were loaded into high-sensitivity glass capillaries and the tryptophan fluorescence was monitored at 330 and 350 nm after excitation at 285 nm. Measurements were made from 25 to 95 °C with a temperature gradient of 1 °C min^−1^. The ratio of fluorescence intensity (350/330 nm) was plotted against temperature, and the first derivative of this curve was used to calculate thermal melting (*T*_m_) values.

### Statistics and reproducibility

Statistical analyses were performed using GraphPad Prism 9. Normally distributed data were compared using two-tailed unpaired *t*-tests whereas non-normally distributed data were compared using two-tailed Mann–Whitney *U*-tests. Differences were considered to be statistically significant when *P* < 0.05. Reported *n* values refer to independent experiments for fluorescence anisotropy, biolayer interferometry analysis and SSA assays. Glycerol gradient sedimentation analysis and size-exclusion chromatography of U2OS nuclear extract recombinant RAD52-OR were repeated independently seven times with similar results. RAD52–ssDNA–RPA pull-down experiments were repeated independently five times with similar results. RAD52 purifications were repeated independently more than 50 times with similar results. RPA purifications were repeated for ten times with similar results. Purifications of RAD52 and RPA mutants were repeated for twice with similar results.

### Reporting summary

Further information on research design is available in the Nature Portfolio Reporting Summary linked to this article.

## Online content

Any methods, additional references, Nature Portfolio reporting summaries, source data, extended data, supplementary information, acknowledgements, peer review information; details of author contributions and competing interests; and statements of data and code availability are available at 10.1038/s41586-024-07347-7.

### Supplementary information

Supplementary Fig. 1 Uncropped blots and gels.

Reporting Summary

## Figures and Tables

**Extended Data Table 1:**
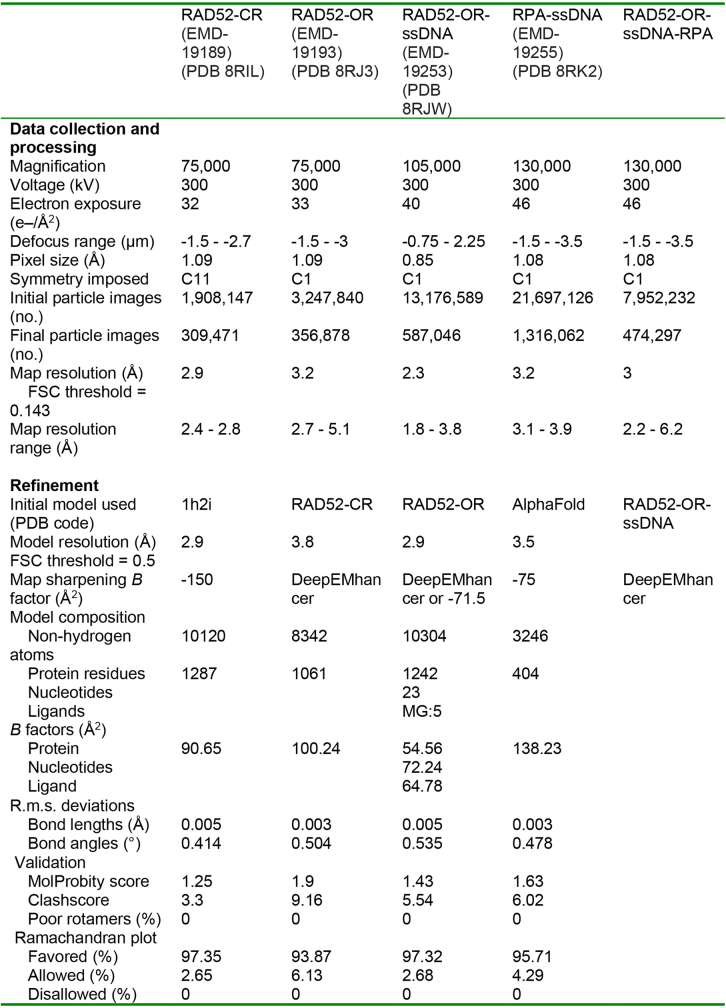
Cryo-EM data collection, refinement and validation statistics

## Data Availability

Cryo-EM density maps and atomic models of RAD52-CR, RAD52-OR, RAD52-OR–ssDNA and RPA–ssDNA have been deposited at the Electron Microscopy Data Bank (EMDB) and PDB under the following accession codes: RAD52-CR (EMD-19189 and 8RIL), RAD52-OR (EMD-19193 and 8RJ3), RAD52-OR–ssDNA (EMD-19253 and 8RJW) and RPA–ssDNA (EMD-19255 and 8RK2). All other data and materials reported here are available on request.
