# Supplementary material for: Mechanism of single-stranded DNA annealing by RAD52–RPA complex
Source: Nature. 2024 Apr 24;629(8012):697–703. doi: 10.1038/s41586-024-07347-7 (PMC11096129; doi:10.1038/s41586-024-07347-7)

nature portfolio

https://doi.org/10.1038/s41586-024-07347-7

Supplementary information

Mechanism of single-stranded DNA annealing by RAD52–RPA complex

In the format provided by the authors and unedited

section used for Fig. 1e

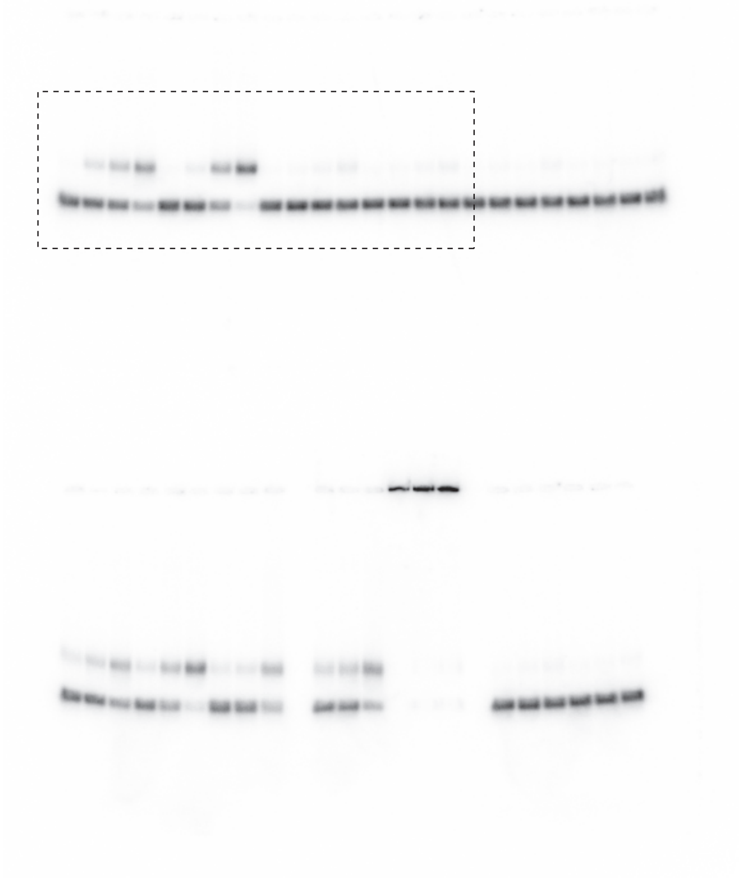

Fig. 1d

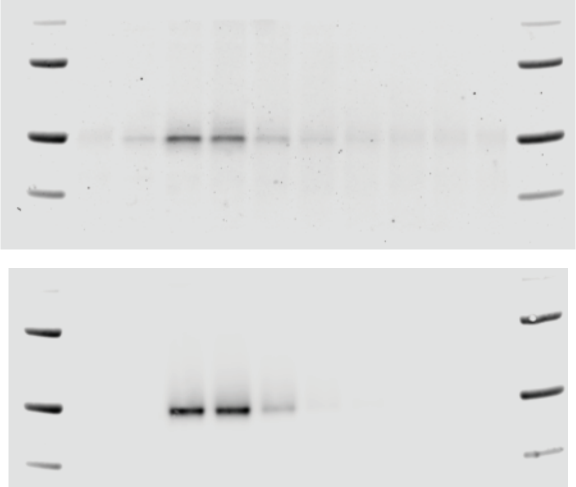

Extended Data Fig. 2c

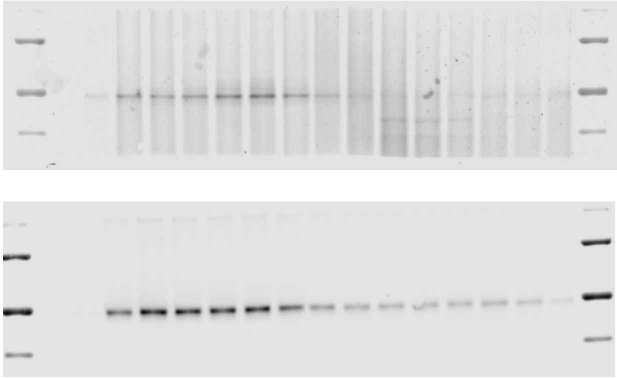

section used for Extended Data Fig. 10c

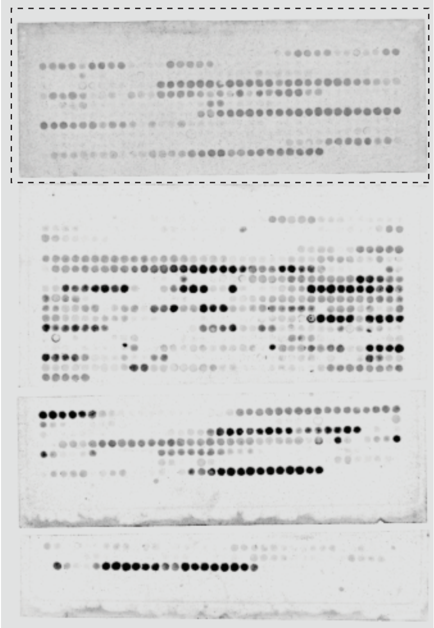

section used for Extended Data Fig. 10d

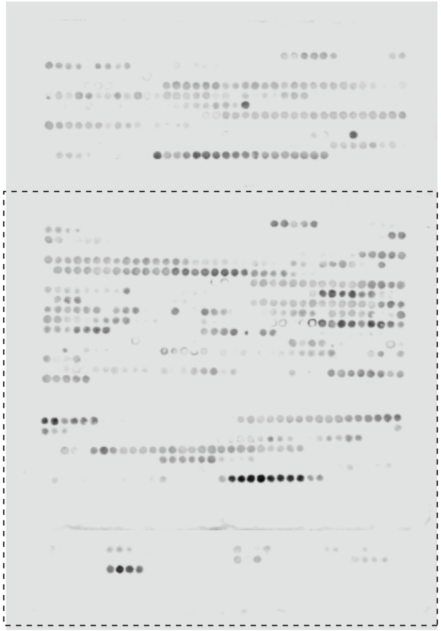

Supplement: Supplementary file 1 — Uncropped blots and gels. [file 41586_2024_7347_MOESM1_ESM.pdf]
