# Supplementary material for: Mechanism of single-stranded DNA annealing by RAD52–RPA complex
Source: Nature. 2024 Apr 24;629(8012):697–703. doi: 10.1038/s41586-024-07347-7 (PMC11096129; doi:10.1038/s41586-024-07347-7)
Supplement: Supplementary file 2 — Reporting Summary [file 41586_2024_7347_MOESM2_ESM.pdf]

nature portfolio

Corresponding author(s): Stephen C. West

Last updated by author(s): 01/02/2024

Reporting Summary

Nature Portfolio wishes to improve the reproducibility of the work that we publish. This form provides structure for consistency and transparency in reporting. For further information on Nature Portfolio policies, see our Editorial Policies and the Editorial Policy Checklist.

Statistics

For all statistical analyses, confirm that the following items are present in the figure legend, table legend, main text, or Methods section.

n/a	Confirmed	
☐	☒	The exact sample size (*n*) for each experimental group/condition, given as a discrete number and unit of measurement
☐	☒	A statement on whether measurements were taken from distinct samples or whether the same sample was measured repeatedly
☐	☒	The statistical test(s) used AND whether they are one- or two-sided<br>*Only common tests should be described solely by name; describe more complex techniques in the Methods section.*
☒	☐	A description of all covariates tested
☒	☐	A description of any assumptions or corrections, such as tests of normality and adjustment for multiple comparisons
☐	☒	A full description of the statistical parameters including central tendency (e.g. means) or other basic estimates (e.g. regression coefficient) AND variation (e.g. standard deviation) or associated estimates of uncertainty (e.g. confidence intervals)
☐	☒	For null hypothesis testing, the test statistic (e.g. *F*, *t*, *r*) with confidence intervals, effect sizes, degrees of freedom and *P* value noted<br>*Give P values as exact values whenever suitable.*
☒	☐	For Bayesian analysis, information on the choice of priors and Markov chain Monte Carlo settings
☒	☐	For hierarchical and complex designs, identification of the appropriate level for tests and full reporting of outcomes
☒	☐	Estimates of effect sizes (e.g. Cohen's *d*, Pearson's *r*), indicating how they were calculated

Our web collection on statistics for biologists contains articles on many of the points above.

Software and code

Policy information about availability of computer code

Data collection	Commercial software available with the respective instruments was used for data collection. These include Chemidoc MP Image Lab Touch Software (version 3.0.1.14, Biorad) for SDS-PAGE gel imaging, ImageStudio (v 5.2, Licor) for imaging of Western blots and peptide arrays, Clariostar BML (Labtech) for fluorescence anisotropy, (v 5.7), EPU (Thermofisher) for cryo-EM data collection, DigitalMicrograph (Gatan) for NS-EM data , UNICORN (Cytiva) for protein purification, AcquireMP (Refeyn) for mass photometry, PR.ThermControl (V2.1.2, Nano Temper) for nanoscale differential scanning fluorometry, Octet BLI Discovery (v12.2, Sartorius) for BLI, ASTRA (v 7.3.2.19, WYATT) and ChromNAV (v 2.03.06; JASCO) for SEC-MALLS.
Data analysis	MARS (Labtech) for blank correction of fluorescence anisotropy data, ImageJ2 (v 2.14) for SSA quantification, MaxEnt1 (Masslynx, Waters) for intact protein mass spectrometry, Octet BLI Analysis (v 12.2, Sartorius) for BLI, PR.Stability ASnalysis (v 1.0.2, Nano temper) for nanoscale differential scanning fluorometry, DiscoverMP (Refeyn) for mass photometry, Proteome Discoverer (v 2.3, ThermoScientific) for XL-MS and AlphaFold2 for protein structure prediction (installed locally).<br>For cryo-EM and NS-EM data analysis: RELION (v 3.1 and 4.0), crYOLO (v 1.7.5), MotionCor2, CTFFIND4, Topaz (v 0.2.5), ChimeraX (v 1.4), COOT (v 0.9.8.7), Phenix (v 1.2), DeepEMhancer, Matchmake<br>ClustalOmega for multiple sequence alignment, ESPript (v 3.0).<br>GraphPad Prism 9 (v 9.2) for statistical analysis and data plotting

For manuscripts utilizing custom algorithms or software that are central to the research but not yet described in published literature, software must be made available to editors and reviewers. We strongly encourage code deposition in a community repository (e.g. GitHub). See the Nature Portfolio guidelines for submitting code & software for further information.

Data

Policy information about availability of data

All manuscripts must include a data availability statement. This statement should provide the following information, where applicable:

- Accession codes, unique identifiers, or web links for publicly available datasets
- A description of any restrictions on data availability
- For clinical datasets or third party data, please ensure that the statement adheres to our policy

The datasets generated during and/or analysed during the current study are included alongside the Article or are available from S.C.W on reasonable request. Cryo-EM density maps and atomic models of RAD52 have been deposited in the Electron Microscopy Data Bank (EMDB) and Protein Database (PDB). Accession codes are as follows: RAD52-CR (EMDB = 19189, PDB = 8RIL), RAD52-OR (EMDB = 19193 , PDB = 8RJ3), RAD52-OR-ssDNA (EMDB = 19253, PDB = 8RJW) and RPA-ssDNA (EMDB = 19255, PDB = 8RK2). All other data is archived at the Francis Crick Institute.

Human research participants

Policy information about studies involving human research participants and Sex and Gender in Research.

Reporting on sex and gender	N/A
Population characteristics	N/A
Recruitment	N/A
Ethics oversight	N/A

Note that full information on the approval of the study protocol must also be provided in the manuscript.

Field-specific reporting

Please select the one below that is the best fit for your research. If you are not sure, read the appropriate sections before making your selection.

☒ Life sciences ☐ Behavioural & social sciences ☐ Ecological, evolutionary & environmental sciences

For a reference copy of the document with all sections, see nature.com/documents/nr-reporting-summary-flat.pdf

Life sciences study design

All studies must disclose on these points even when the disclosure is negative.

Sample size	Wherever quantification is provided, a minimum 3 independent experiments were carried out to perform statistical analyses as indicated in figure legends as n.
Data exclusions	In SSA assays, some samples remained in the wells because of incomplete deproteinisation. These samples were not quantified and excluded from the datasets.<br>In cryo-EM processing, micrographs with CTF outliers (often due to presence of contaminating ice or large areas of foil) were removed from analyses. Picked particles that did not align in 2D or 3D were removed from further analysis. For NS-EM, all datasets went through two rounds of 2D classification.
Replication	To ensure the replication, each experiment was performed at least three times in exact condition on different days. All attempts were successful. The cryo-EM structures obtained from 300 kV Krios were also observed in other lower resolution datasets obtained on the 200 kV TALOS (data not shown).
Randomization	For calculation of resolution in cryo-EM maps, FSCs were calculated using independent halves of the datasets, into which the particles were randomly allocated. In addition, Topaz particle picking models were trained on a random subset of particles. Randomization is not relevant to the in vitro analysis of recombinant proteins, and therefore was not used in this study.
Blinding	Blinding is not necessary for analysis of recombinant proteins by cryoelectron microscopy, single molecule methods or biochemistry. The researcher needs to know the protein sample used to conduct the experiments performed, and therefore blinding is not appropriate.

Reporting for specific materials, systems and methods

We require information from authors about some types of materials, experimental systems and methods used in many studies. Here, indicate whether each material, system or method listed is relevant to your study. If you are not sure if a list item applies to your research, read the appropriate section before selecting a response.

Materials & experimental systems

n/a	Involved in the study
☐	☒ Antibodies
☐	☒ Eukaryotic cell lines
☒	☐ Palaeontology and archaeology
☒	☐ Animals and other organisms
☒	☐ Clinical data
☒	☐ Dual use research of concern

Methods

n/a	Involved in the study
☒	☐ ChIP-seq
☒	☐ Flow cytometry
☒	☐ MRI-based neuroimaging

Antibodies

Antibodies used	For Western blotting, we used the following antibodies. RAD52 (Rabbit monoclonal, 1:500, ab124971, Abcam) and 6xHis (Mouse monoclonal, 1:1000, 631212, TakaRa) . Membranes were incubated with Alexa Fluor Plus 800 anti-rabbit secondary antibody (1:2000, Invitrogen, A32735) or Alexa Fluor Plus 800 anti-mouse secondary antibody (1:2000, Invitrogen, A32730) .
Validation	Antibodies in this study are only used for immunoblotting against recombinant proteins.

Eukaryotic cell lines

Policy information about cell lines and Sex and Gender in Research

Cell line source(s)	U2OS and Sf9 cells are available from The Francis Crick Institute, London, UK upon request.
Authentication	U2OS and Sf9 cells have been authenticated and confirmed to be the correct cell line.
Mycoplasma contamination	U2OS and Sf9 cells have tested negative for mycoplasma
Commonly misidentified lines (See ICLAC register)	No commonly misidentified cell lines have been used in this study.
